# Repeated stress gradually impairs auditory processing and perception

**DOI:** 10.1371/journal.pbio.3003012

**Published:** 2025-02-11

**Authors:** Ghattas Bisharat, Ekaterina Kaganovski, Hila Sapir, Anita Temnogorod, Tal Levy, Jennifer Resnik

**Affiliations:** 1 Department of Life Sciences, Ben-Gurion University of the Negev, Beer Sheva, Israel; 2 Zelman Center for Brian Science Research, Ben-Gurion University of the Negev, Beer Sheva, Israel; University College London, UNITED KINGDOM OF GREAT BRITAIN AND NORTHERN IRELAND

## Abstract

Repetitive stress, a common feature of modern life, is a major risk factor for psychiatric and sensory disorders. Despite the prevalence of perceptual abnormalities in these disorders, little is known about how repetitive stress affects sensory processing and perception. Here, we combine repetitive stress in mice, longitudinal measurement of cortical activity, and auditory-guided behaviors to test if sound processing and perception of neutral sounds in adults are modulated by repetitive stress. We found that repetitive stress alters sound processing, increasing spontaneous cortical activity while dampening sound-evoked responses in pyramidal and PV cells and heightening sound-evoked responses in SST cells. These alterations in auditory processing culminated in perceptual shifts, particularly a reduction in loudness perception. Additionally, our work reveals that the impact of stress on perception evolves gradually as the stressor persists over time, emphasizing the dynamic and evolving nature of this mechanism. Our findings provide insight into a possible mechanism by which repetitive stress alters sensory processing and behavior, challenging the idea that stress primarily modulates emotionally charged stimuli.

## Abstract 2

How does repetitive stress impact sensory processing and perception? This study reveals a pathway through which repetitive stress modifies basic sensory processing and perception in mice, showing how repetitive stress alters sound processing through progressive changes that intensify with continued exposure.

## Introduction

Repetitive stress exerts a profound impact on mental health, acting as a catalyst for psychiatric disorders and sensory impairments [1,2]. This sustained stress triggers a cascade of adaptive reactions within the central and peripheral systems, orchestrating behavioral adjustments to cope with the altered internal state [3]. In contrast to fear or acute stress, which elicit rapid and temporary defensive responses to a specific stimulus, a state of chronic or repetitive stress persists after the initial sensory trigger has dissipated [4,5]. While research on chronic and repetitive stress has made significant progress in understanding its effects on complex cognitive processes like learning, memory, and decision-making [3,6–12], there remains a notable gap in our understanding of its influence on fundamental cortical functions, such as sensory processing.

Our sensory experiences are intricately linked to our internal state, causing the perception of a given stimulus to fluctuate significantly based on our internal conditions. For instance, the sound of a doorbell may seem louder when we are stressed, and odors can appear more aversive when we are anxious [13]. Yet, does chronic and repetitive stress actually alter our sensory processing and perception?

Previous studies have shown that chronic and repetitive stress can modulate the perception of stimuli with preexisting positive or negative connotations. For example, chronic stress heightens sensitivity to pain through increased spinal neuroinflammation [14] and elevated synchronous activity between the anterior cingulate cortex and amygdala regions [15]. It impacts odor preferences by diminishing adult neurogenesis in the olfactory bulb, affecting attraction to pleasant and unpleasant odors [16]. Despite these significant findings, the influence of chronic and repetitive stress on sensory processing and perception, especially concerning neutral everyday stimuli with no inherent positive or negative associations, remains largely unexplored. Some evidence indicates that physiologically or metabolically chronic stress can affect sensory-guided behaviors, such as the ability to detect new odors [17] or new textures [18] and odor investigation time [16]. However, separating the sensory component from the memory component of these behaviors has proven challenging. There are also indications of changes in primary sensory areas like the piriform cortex, where c-Fos expression increases in response to chronic stress [19]. Collectively, these findings suggest that chronic and repetitive stress could modulate primary sensory areas, potentially influencing perception through alterations in their activity. However, direct measurements of activity changes in the piriform cortex or any other primary sensory areas during chronic and repetitive stress are currently lacking.

To investigate whether and how chronic and repetitive stress modulates the activity of primary sensory areas, we specifically focused on assessing changes in the activity in the auditory cortex. This selection was guided by several factors. Firstly, previous research indicates that early life stress can alter sound processing, leading to reduced sensitivity to gaps and diminished auditory evoked potentials [20]. Secondly, the auditory cortex is known to be susceptible to modulation through fear conditioning and limbic system activity, which is implicated in the stress response [21]. Additionally, the auditory cortex is not solely dictated by auditory features of the sensory input; rather, its activity reflects a complex interplay between external stimuli and context [22–30]. Together, these findings support the hypothesis that the auditory cortex could be influenced by a change in the overall state of the animal, such as repetitive stress.

## Results

### Physiological and behavioral evidence of stress

**Fig 1:**
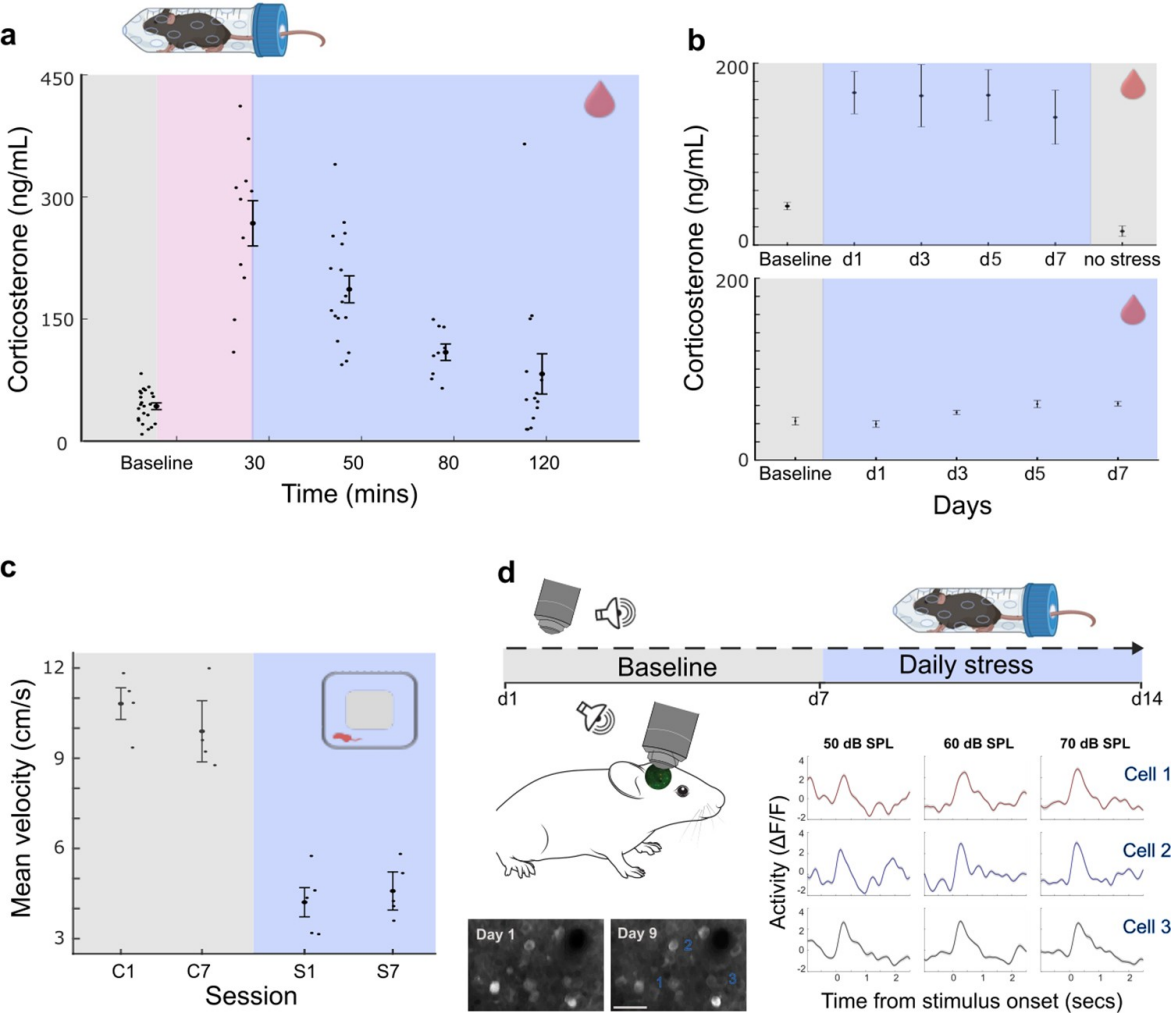
Physiological and behavioral evidence of stress. (a) Corticosterone levels at different time points, before and after 30 min of restraint stress. Corticosterone levels increased following restraint stress (ANOVA F = 28.27, *p* = 6.4 × 10^−14^). Values represent mean ± SE. (b) Top: Corticosterone levels before, during 7 days of restraint stress, and 2 days after restraint stress was stopped. The measurements were pooled from different time points (30 to 120 min after restraint stress). Corticosterone levels increased during the week of repeated stress compared to baseline (ANOVA F = 7.7, *p* = 6.3 × 10^−14^). Bottom: Slight increase in pre-restraint corticosterone levels (before the mouse entered the 50 ml tube) as the stressor persists over time (ANOVA F = 3.04, *p* = 0.028). Values represent mean ± SE. (c) Movement velocity during 20-min open-field sessions in control (marked as C) and mice undergoing repeated stress (marked as S). Mice were tested on both the initial and seventh day of stress, while control mice followed the same protocol but without experiencing restraint stress (*N* = 5 and 4, accordingly). The mice exhibited reduced activity levels under repeated stress conditions. Notably, there was no discernible distinction in their behavior between the first and seventh day of testing, indicating a lack of adaptation over time (2-way ANOVA, condition F = 157.7, *p* = 2.1 × 10^−08^, session number F = 0.26, *p* = 0.61, interaction F = 1.49, *p* = 0.24). Values represent mean ± SE. (d) Left: Schematics of two-photon imaging during baseline and repetitive stress conditions. In repetitive stress sessions, the mice were placed in a 50 ml tube for 30 min to achieve mild stress. The imaging session started directly after the restraint. Individual cells were tracked over imaging days. Shown are examples of 2 imaging planes on day 1 and day 9 (scale bar, 50 μm) and the noise-evoked responses of 3 exemplar cells (mean ± SE). (e) Source data for this figure can be found at: https://www.ebi.ac.uk/biostudies/studies/S-BSST1689754.

Repeated stress exposure can induce chronic stress, although certain stressors may trigger habituation [31]. To address this issue, we tested whether half an hour of daily restraint stress [32] could cause mild chronic stress without habituation. We measured physiological and behavioral stress markers in mice, in baseline conditions, and during 7 days of daily restraint stress. As expected, we observed elevated corticosterone levels following restraint stress [33], which stayed high for over an hour (Fig 1A, 1-way ANOVA F = 28.27, *p* = 6.4 × 10^−14^). As the stressor persisted over time, we noticed a slight increase in pre-restraint corticosterone levels, consistent with chronic stress [34] (Fig 1B, bottom, 1-way ANOVA F = 3.04, *p* = 0.028). Importantly, we did not observe any signs of habituation when repeating the restraint stress protocol for a week (Fig 1B, top). During the open-field test, a well-established behavioral measure of stress and anxiety [35], mice displayed decreased activity levels throughout the week of daily stress when compared to the control group [36,37]. Control mice followed the same protocol but without experiencing restraint stress. This reduced activity remained consistent after 7 days of restraint stress (Fig 1C, 2-way ANOVA, condition F = 157.7, *p* = 2.1 × 10^−08^, session number F = 0.26, *p* = 0.61, interaction F = 1.49, *p* = 0.24). To assess whether this approach disrupted the regulation of the stress response, potentially affecting learning and memory [38], we compared glucocorticoid receptor (GR) expression in the primary auditory cortex of a new group of mice subjected to a week of repeated restraint stress (S1A Fig, *N* = 3) to control mice that did not undergo restraint stress (*N* = 3). Our analysis revealed no significant difference in GR expression between the 2 groups (S1A Fig, *t* test *p* = 0.9) indicating that mild restraint stress did not result in GR dysregulation. Additionally, we examined whether repeated restraint stress impacted the auditory periphery by measuring auditory brainstem responses (ABR). We observed no changes in wave 1 amplitude or ABR threshold (S1B Fig), suggesting that auditory function remained intact. These findings suggest that during daily restraint stress the mice sustained a balanced stress response with no peripheral auditory damage. Collectively, our results demonstrate that daily restraint stress induces a consistent stress response without signs of habituation.

### Repetitive stress induces a decrease in sound-evoked activity

Earlier studies have identified alterations in both excitatory and inhibitory activity in the prefrontal cortex, amygdala, and hippocampus during chronic and repetitive stress, frequently entailing changes in the structure, molecular expression, and activity of interneurons, notably PV cells [39–43]. Therefore, to assess the impact of repetitive stress on sensory processing and perception, we simultaneously track changes in the activity of parvalbumin-expressing (PV) and putative pyramidal neurons (PPys). We performed chronic two-photon calcium imaging from the primary auditory cortex (ACtx) of awake, head-fixed mice that expressed tdTomato in PV neurons and GCaMP6s non-selectively, in L2/3 neurons (Fig 1D). Expressing the stable tdTomato fluorophore in PV neurons facilitated motion correction and chronic tracking of individual neurons for several days [44] and allowed us to analyze GCaMP signals coming from PV neurons separately from surrounding PPys [45].

The observed decrease in c-fos expression within the primary somatosensory cortex during chronic stress [46] led us to hypothesize that there could be a decrease in sensory-evoked activity during stressful states. Such a decrease in responsiveness to sound stimuli might serve as an adaptive mechanism, conserving energy resources for the prioritized encoding of more immediate and pressing needs amid stressful periods. Conversely, heightened states, like chronic stress, might augment sound-evoked activity, potentially signaling danger. To distinguish between these possibilities, we initially examined cortical responses during repetitive stress. We presented a white noise stimulus—devoided of predictable patterns (Fig 1d)—at different intensities every other day, to keep the imaging sessions brief and prevent extended exposure and photobleaching, as well as to prevent habituation to the stimuli. Importantly, all sounds were neutral, devoid of any association with the stress-inducing cause, in contrast to fear conditioning paradigms. We presented the different stimuli at intensities and durations carefully chosen to ensure that they would not become stressors in and of themselves. This approach allowed us to test if repetitive stress influences cortical responses to neutral sounds, not linked to an aversive outcome.

**Fig 2:**
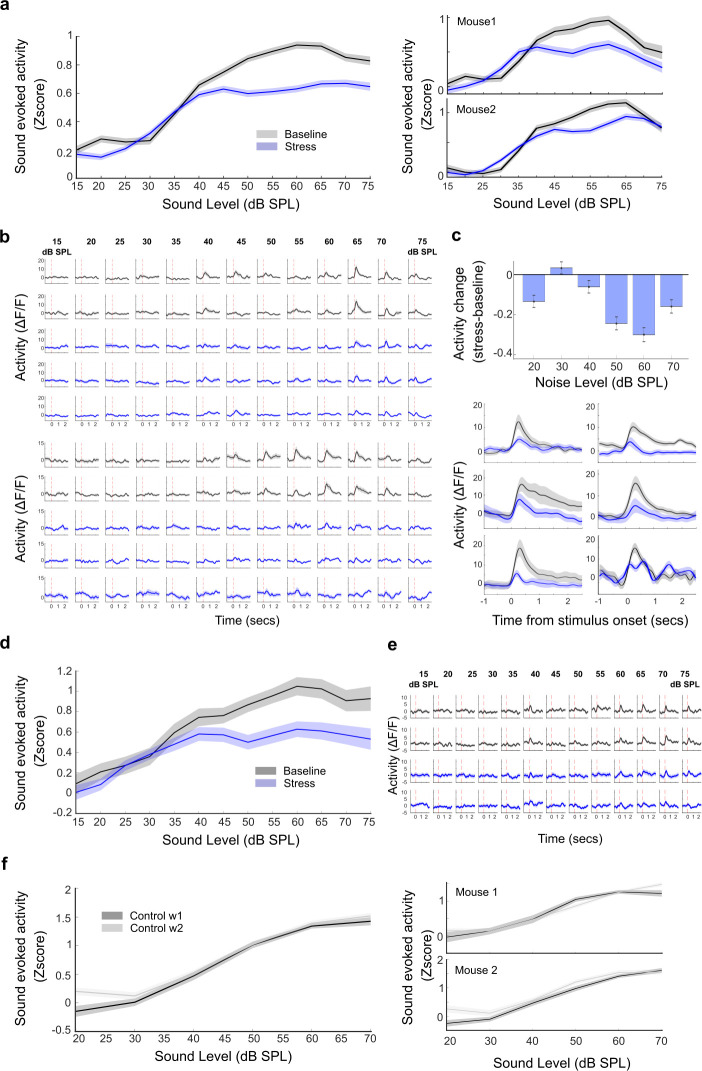
Repetitive stress induces a decrease in sound-evoked activity. (a) Left: noise-evoked activity rates at different noise intensities for chronically tracked PPys cells in baseline and repeated stress conditions (*N* = 5 mice, *n* = 285 neurons, mean ± SE). Activity rates decreased during repeated stress compared to baseline (2-way ANOVA, condition F = 185.6, *p* = 4.8 × 10^−42^, condition: intensity interaction F = 10.37, *p* = 9.3 × 10^−21^, nested ANOVA (mouse nested within session), condition F = 174, *p* = 1.5 × 10^−39^, condition: intensity interaction F = 12.7, *p* = 2 × 10^−26^, post hoc for each level baseline versus repetitive stress *p* < 0.01 for all levels above 50 dB, all Bonferroni corrected). Right: noise-evoked activity rates in 2 exemplar mice (mean ± SE). (b) Example of ΔF/F traces of 2 PPys tracked cells, recorded in response to noise presented at sound intensities ranging from 15 to 75 dB SPL in 2 baseline and 3 repeated stress sessions. Marked at time = 0 is the onset of the 100-ms white noise. (c) Top: Mean activity change, mean stress activity minus mean baseline activity, calculated per cell at different noise intensities. The reduction in activity was particularly striking at moderate sound intensities (mean ± SE, 1-way ANOVA, F = 14.24, *p* = 1.0 × 10^−13^ and *t* test for each level: 20 dB *p* = 8.4 × 10^−05^, 30 dB *p* = 1, 40 dB *p* = 0.350 dB *p* = 4.9 × 10^−12^, 60 dB *p* = 2.4 × 10^−15^, 70 dB *p* = 1.5 × 10^−05^ all corrected for multiple comparisons. Bottom: 50 dB noise evoked activity of 6 exemplar PPys cells during baseline and stressful states. (d) Noise-evoked activity rates at different noise intensities for chronically tracked PV cells in baseline and repeated stress conditions (*N* = 5 mice, *n* = 31 neurons, mean ± SE). Activity rates decreased during repeated stress compared to baseline (2-way ANOVA, condition F = 49.6, *p* = 2.6 × 10^−12^, condition: intensity interaction F = 1.94, *p* = 0.02, nested ANOVA (mouse nested within session), F = 56.5, *p* = 8.8 × 10^−14^, condition: intensity interaction F = 3.5, *p* = 3.5 × 10^−05^). (e) Example of ΔF/F traces of one tracked PV cell, recorded in response to noise presented at sound intensities ranging from 15 to 75 dB SPL in 2 baseline and 2 repeated stress sessions. Marked at time = 0 is the onset of the 100-ms white noise. (f) A second group of mice followed the same procedures but without experiencing daily restraint stress (*N* = 3 mice, *n* = 123 neurons). These mice exhibited a minimal change in noise-evoked PPy activity when comparing the first and second week of imaging (2-way ANOVA, F = 1.79, *p* = 0.11, post hoc baseline w1 50 dB: baseline w2 50 dB *p* = 1 Bonferroni corrected, nested ANOVA (mouse nested within session) F = 1.76, *p* = 0.12, mean ± SE). Right: mean PPy activity of 2 representative mice. Source data for this figure can be found at: https://www.ebi.ac.uk/biostudies/studies/S-BSST1689754.

Using each cell as its own control, we calculated the daily changes in sound-evoked activity in baseline and stressful states. When we directly tested the cortical activity, we found that chronically tracked PPys neurons (*N* = 5 mice, *n* = 285 tracked neurons) showed an intensity-specific reduction in noise-evoked activity during repetitive stress (Fig 2A and 2B, nested 2-way ANOVA, condition F = 174, *p* = 1.5 × 10^−39^, condition: intensity interaction F = 12.7, *p* = 2 × 10^−26^). The reduction in activity was particularly striking at moderate sound intensities, whereas the response to loud noise intensities was more similar to baseline values (Fig 2C, 1-way ANOVA, intensities: F = 14.24, *p* = 1.0 × 10^−13^). We also found, during repetitive stress, a small reduction in the percentage of sound-responsive cells (calculated from all imaged cells, S2A Fig, left, *t* test, *p* = 0.02). We observed a similar reduction in activity when we examined the activity of PV cells (*N* = 5 mice, *n* = 31 tracked neurons) in baseline and repetitive stress conditions (Fig 2D and 2E, 2-way nested ANOVA condition, F = 56.5, *p* = 8.8 × 10^−14^, condition: intensity interaction F = 3.5, *p* = 3.5 × 10^−05^). However, there was no significant change in the percentage of sound-responsive PV cells (S2A Fig, right, *t* test, *p* = 0.19).

The lack of change in response to soft sound intensities and the return of response strength to high sound intensities to baseline values suggest that the observed changes were not a result of habituation. To confirm this, we tested a second group of mice following the same procedures but without experiencing daily restraint stress (Fig 2F, *N* = 3 mice, *n* = 123 pyramidal neurons). These mice exhibited minimal change in noise-evoked activity when comparing the first and second week of imaging (Fig 2F, nested 2-way ANOVA, F = 1.76, *p* = 0.12, post hoc baseline w1 50 dB: baseline w2 50 dB, *p* = 1 Bonferroni corrected). This finding underscores that the diminished activity observed in the initial group of mice was primarily attributable to repetitive stress rather than to our experimental paradigm.

### Repetitive stress causes a reduction in the neural contrast between pre-and post-sound periods

**Fig 3:**
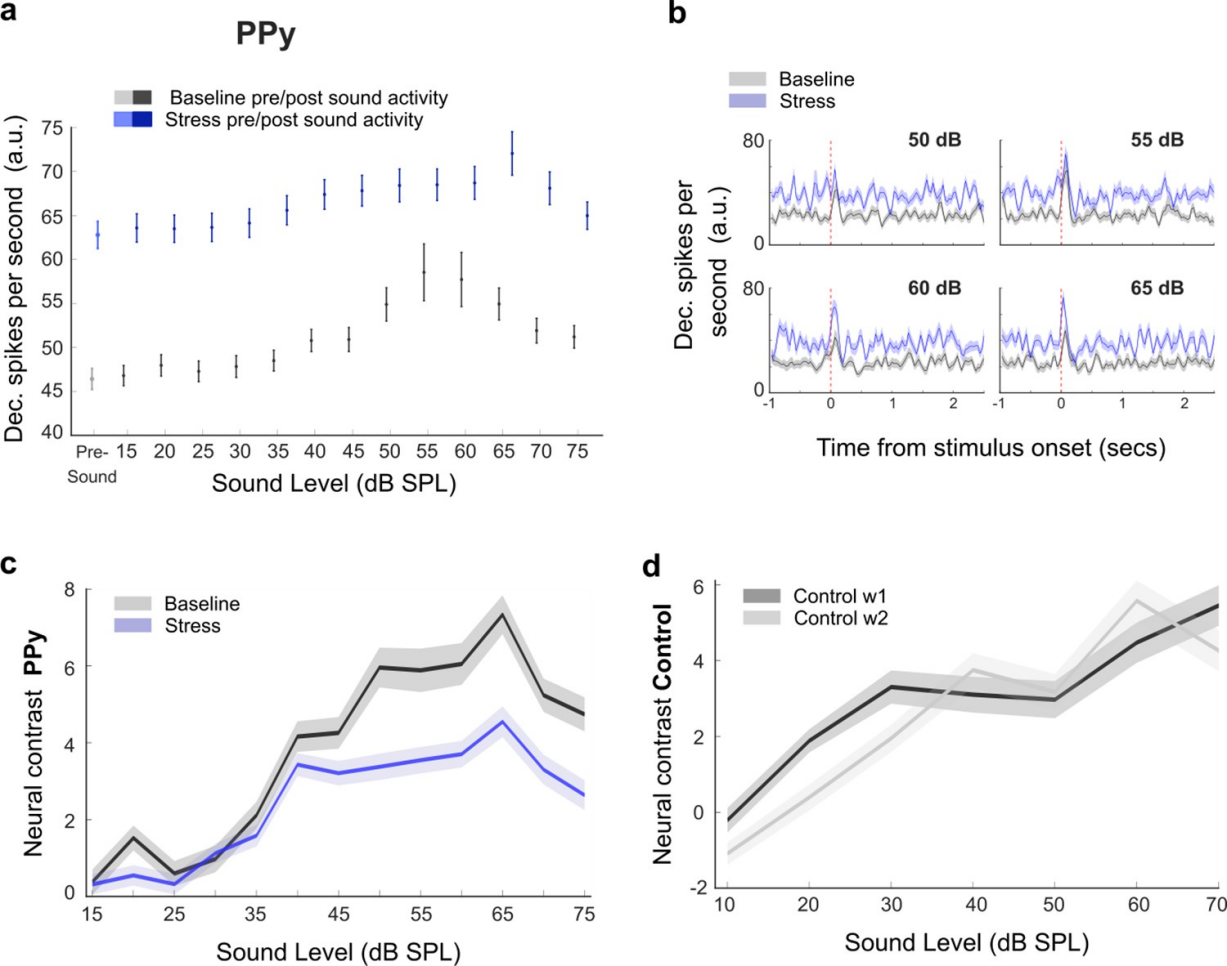
Repetitive stress causes a reduction in neural contrast. (a) Mean pre- and post-sound activity presented as deconvolved spikes. Pre- and post-sound activity increased during repeated stress (2-way ANOVA condition F = 413, *p* = 1.4 × 10−^263^, nested ANOVA (mouse nested within session) F = 668.5, *p* < 2.2 × 10^−16^, mean ± SE). (b) Example of deconvolved spike traces of a tracked PPy cell, recorded in response to noise presented at sound 50-55-60-65 dB SPL in baseline and stress sessions. Marked at time = 0 is the onset of the 100-ms white noise. (c) Neural contrast calculates as (post-sound activity—pre-sound activity)/(post-sound activity + pre-sound activity)*100. The neural contrast decreased during repeated stress (2-way ANOVA, condition F = 84.4, *p* = 4.3 × 10^−20^, condition: intensity interaction F = 3.7, *P* = 8.3 × 10^−06^, nested ANOVA (mouse nested within session), condition F = 50.7, *p* = 1 × 10^−12^, condition: intensity interaction F = 7.7, *P* = 3.1 × 10^−105^, mean ± SE). (d) Neural contrast for the control group. Control mice followed the same protocol but without experiencing restraint stress. There was no change in neural contrast between the first and second week of imaging (2-way ANOVA, condition F = 2.9, *p* = 0.08, nested ANOVA (mouse nested within session), condition F = 0.7, *p* = 0.3, mean ± SE). Source data for this figure can be found at: https://www.ebi.ac.uk/biostudies/studies/S-BSST1689754.

Our findings thus far indicate that repetitive stress leads to a reduction in noise-evoked responses, particularly evident at mid-intensities. Since changes in sound-evoked activity may stem from alterations in pre-sound or post-sound activity, or from shifts in their relationship [25,47–49], we examined the modulation of pre-sound activity and post-sound activity individually during repetitive stress. Using each cell as its own control, we examined the daily changes in activity in baseline and stressful days. For a more detailed and accurate examination of pre-sound activity, we examined the cortical activity measured as deconvolved spikes [44] (Fig 3A and 3B). We found that chronically tracked PPys neurons showed a general increase in activity during repetitive stress. We observed this increase in activity both in the pre- and post-sound periods (Fig 3A, nested 2-way ANOVA, condition F = 668.5, *p* < 2.2 × 10^−16^). This increase was not merely additive; instead, the relative increase in post-sound activity seemed to vary depending on the intensity of the noise. To test if this was the case, we calculated the neural contrast between the pre- and post-sound periods and assessed its modulation by repetitive stress. Indeed, we found that the neural contrast between the pre- and post-sound periods was smaller for mid- and high-sound intensities during repetitive stress (Fig 3C, nested 2-way ANOVA, condition F = 50.7, *p* = 1 × 10^−12^), but not in the control group (Fig 3D, nested 2-way ANOVA, condition F = 0.7, *p* = 0.3). This reduction in the contrast between pre- and post-sound activity explains the stress-induced reduction in sound-evoked activity that we found above (Fig 2).

**Fig 4:**
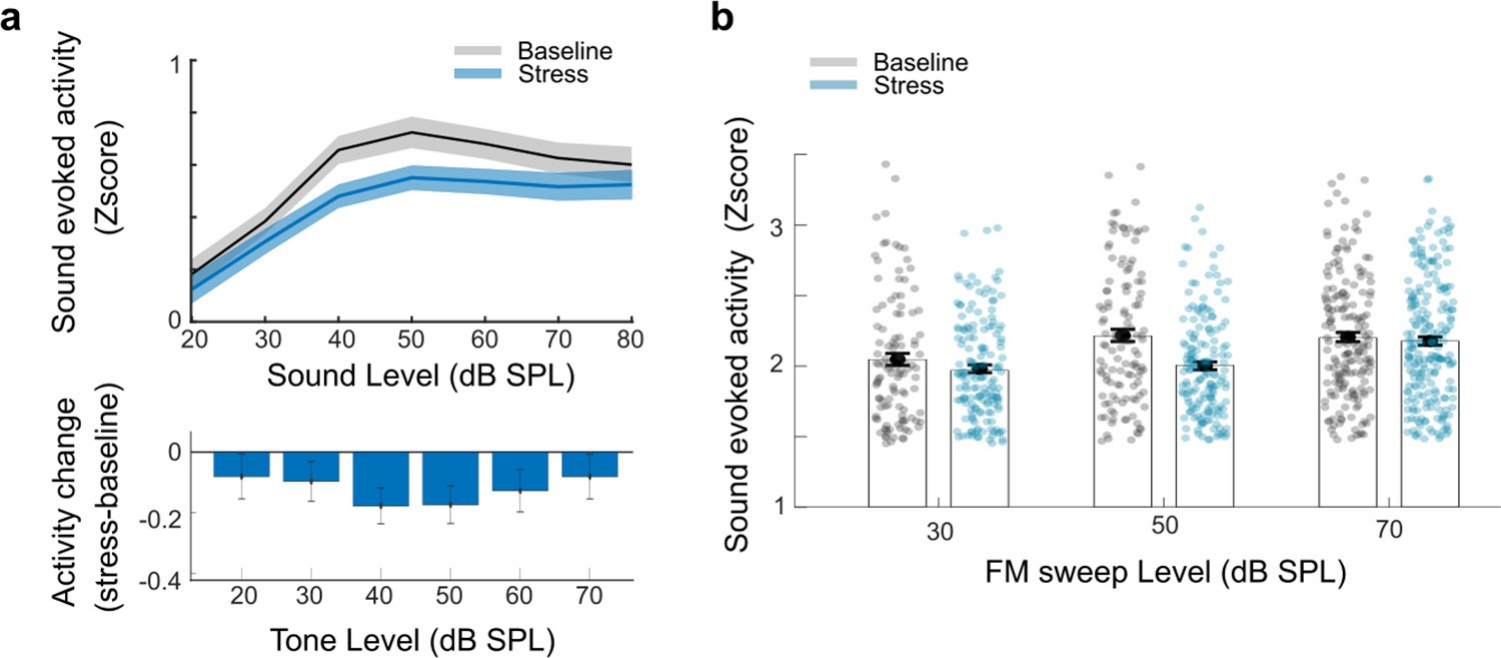
Alterations in tone and FM sweeps evoked activity during repetitive stress. (a) Pure tone-evoked activity for chronically tracked cells at different sound intensities during baseline and repeated stress conditions. Activity rates decreased at moderate sound intensities, while activity at high intensities remained comparable to baseline intensities (Fig 2D, 2-way ANOVA, condition F = 16.1, *p* = 6.02 × 10^−05^, nested ANOVA (mouse nested within session) F = 16.1, *p* = 5.9 × 10^−05^, mean ± SE). Bottom: Mean activity change calculated per cell at different sound intensities. The reduction in activity was particularly striking at moderate sound intensities (*t* test for 40 dB *p* = 0.003, 50 dB *p* = 0.006), whereas activity at higher and lower intensities remained on par with baseline values (*t* test for 20 dB = 0.2, 30 dB = 0.14, 60 dB = 0.07, 70 dB *p* = 0.27). Values represent mean ± SE. (b) FM sweep-evoked activity for chronically tracked cells at different sound intensities during baseline and repeated stress conditions. There was a decrease in activity during repetitive stress (mean ± SE, 2-way ANOVA condition F = 13.9, *p* = 2.03 × 10^−04^, condition: intensity interaction F = 4.3, *p* = 0.01, nested ANOVA (mouse nested within session) F = 2.1, *p* = 0.001 condition: intensity interaction F = 2.1, *p* = 0.001), especially at moderate sound intensities (post hoc 50 dB *p* = 2.36 × 10^−04^, but not at lower 30 dB *p* = 1 or higher intensities 70 dB *p* = 1 Bonferroni corrected). Source data for this figure can be found at: https://www.ebi.ac.uk/biostudies/studies/S-BSST1689754.

Our current findings suggest that repetitive stress results in a relative decrease in noise-evoked responses. One could imagine that this reduction in activity could be specific to white noise, given its potential to mask relevant sensory information during periods of stress. Alternatively, this reduction of activity could represent a broader mechanism affecting responses to all sound stimuli. To distinguish between these possibilities, we tested the change in cortical responses during repetitive stress to different sound stimuli. We played the mice a frequency-modulated (FM) sweep, a fundamental component of complex communication signals in speech, music, and conspecific vocalization, and a simpler auditory stimulus devoid of any unpredictability or complexity—a 12 kHz pure tone, both at different sound intensities. We expected that if repetitive stress affected the response to all auditory stimuli, we would observe a reduction in activity to the pure tones and FM sweeps as well. If the phenomenon was specific to noise, we would not observe such a difference. Analysis of the tone-evoked activity revealed a reduction in activity at moderate sound intensities, while activity at high intensities remained comparable to baseline levels (Fig 4A, top 2-way nested ANOVA, condition: F = 16.1, *p* = 5.9 × 10^−05^, bottom: *t* test 50 dB *p* = 0.006, 70 dB *p* = 0.27). Similarly, analysis of the FM sweep-evoked activity demonstrated a decrease during repetitive stress (Fig 4B, nested 2-way ANOVA condition: F = 2.1, *p* = 0.001, condition: intensity interaction F = 2.1, *p* = 0.001), especially at moderate sound intensities (post hoc 50 dB SPL baseline versus stress *p* = 2.36 × 10^−04^, 70 dB SPL baseline versus stress *p* = 0.9 both Bonferroni corrected). Taken together, our results indicate that this is a general mechanism: Repetitive stress induces a reduction in sound-evoked cortical activity in an intensity-dependent manner.

### Divergent modulation of inhibitory cells during repetitive stress

Our findings indicate that repetitive stress leads to an increase in both pre- and post-sound activity, but this increase is unbalanced, resulting in reduced sound-evoked responses in both PPy and PV cells. What could be driving this shift in activity? One possibility is that the balance between inhibition and excitation remains unchanged, leading to a general reduction in sound-evoked activity in the ACtx during repetitive stress. Another possibility is that altered activity in a distinct inhibitory cell type may contribute to the reduced sound-evoked responses in PPy cells. Chronic stress has been shown to increase somatostatin expression in the dorsal hippocampus of male rats [40] and to alter the structure of somatostatin-expressing cells by increasing dendritic complexity in adult male mice [50]. This raises the possibility that changes in the activity of somatostatin-expressing inhibitory (SST) cells could underlie the observed reduction in sound evoked-activity in PPy and PV.

**Fig 5:**
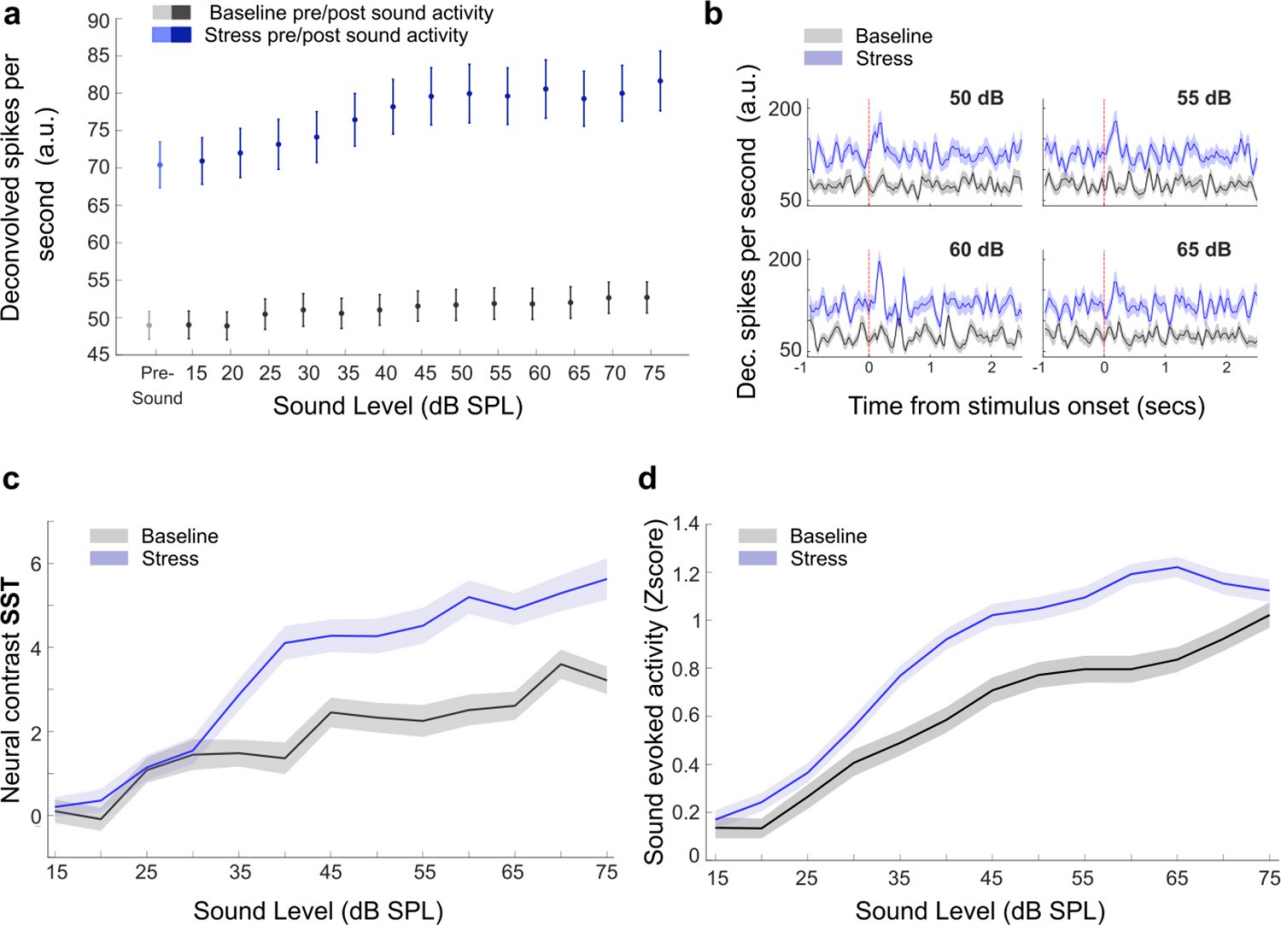
Divergent modulation of inhibitory cells during repetitive stress. (a) Mean noise-evoked activity (deconvolved spikes) for different noise intensities in the baseline (gray) and repetitive stress (blue) for chronically tracked SST cells (*N* = 4 mice, *n* = 121 tracked cells). The activity was averaged over a 300 ms period before (soft) and after the sound (dark colors). There was an increase in pre- and post-sound activity during repeated stress (mean ± SE, 3-way ANOVA, condition F = 805, *p* = 1.5 × 10^−171^ nested ANOVA (mouse nested within session) F = 853, *p* = 2.4 × 10^−181^). (b) Example of deconvolved spike traces of a tracked SST cell, recorded in response to noise presented at sound 50-55-60-65 dB SPL in baseline and stress sessions. Marked at time = 0 is the onset of the 100-ms white noise. (c) Mean neural contrast between the pre-sound and post-sound windows for tracked SST cells in baseline and repetitive stress. The neural contrast was calculated as (post sound activity—pre sound activity)/(post sound activity + pre sound activity)*100 per cell. There was an increase in the neural contrast during repeated stress (mean ± SE, 2-way ANOVA, condition F = 108.4, *p* = 3.5 × 10^−25^, nested ANOVA (mouse nested within session) F = 116.3, *p* = 7 × 10^−27^). (d) The activity per SST cell was normalized (z-score) before calculating the mean noise-evoked activity. There was an increase in sound-evoked activity during repeated stress (mean ± SE, 2-way ANOVA, condition F = 150.4, *p* = 3.4 × 10^−34^, nested ANOVA (mouse nested within session) F = 125.6, *p* = 7 × 10^−29^). Source data for this figure can be found at: https://www.ebi.ac.uk/biostudies/studies/S-BSST1689754.

To differentiate between these possibilities, we tracked the activity of SST cells in a new group of mice during the same protocol of baseline and repetitive restraint stress. In this experiment, we performed chronic two-photon calcium imaging from the ACtx of awake, head-fixed mice that selectively expressed GCaMP8f in SST L2/3 neurons. Using each cell as its own control, we calculated the daily changes in sound-evoked activity across conditions. Chronically tracked SST neurons (Fig 5, *N* = 4 mice, *n* = 121 tracked neurons) also showed increased pre- and post-sound activity (Fig 5A and 5B, nested 3-way ANOVA, condition F = 853, *p* = 2.4 × 10^−181^). However, unlike PPy and PV cells, SST cells showed a greater difference between pre- and post-sound activity under repetitive stress (Fig 5C, nested 2-way ANOVA, condition F = 116.3, *p* = 7 × 10^−27^). This increase in activity difference led to an increase in noise-evoked activity (Fig 5D, nested 2-way ANOVA, condition F = 125.6, *p* = 7 × 10^−29^), without a significant increase in the percentage of sound-responsive cells (S2B Fig, *t* test *p* = 0.1). These results indicate that SST cells are differently modulated under repetitive stress, and their increased activity may contribute to reduced sound-evoked responses in PPy and PV cells.

To ensure that the slight differences in cell tracking strategy used in the SST experiment were not biasing our results, we applied the same viral approach to a new group of mice to track PV cells (S3 Fig, *N* = 2 mice, *n* = 81). We conducted chronic two-photon calcium imaging in the ACtx of mice selectively expressing GCaMP8f in PV L2/3 neurons. Consistent with our prior observations when tracking PV cells expressing tdTomato (Fig 2D), we found an increase in both pre- and post-sound activity, along with a reduction in neural contrast between these periods (S3 Fig). These results indicate that repeated stress enhances sound-evoked activity in SST cells, while suppressing sound-evoked activity in PV and PPy cells. This differential modulation across cell subpopulations may diminish the prominence of sound-related signals transmitted to downstream regions during repeated stress.

### The effect of stress on sound processing increases with repeated exposure

**Fig 6:**
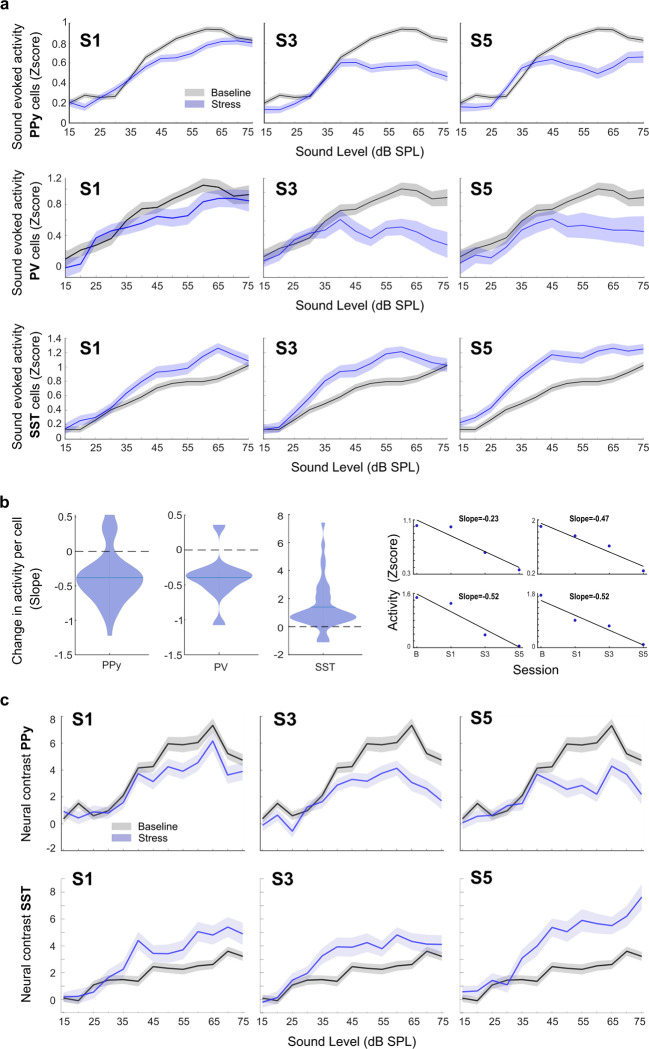
The effect of stress on sound processing increases with repeated exposure. (a) Change of noise-evoked activity over time. Comparison of noise-evoked activity after a day, 3 days, and 5 days of stress to baseline. Middle-intensity sound-evoked activity decreased as stress became chronic for PPy cells (2-way ANOVA, condition S1 F = 35.3, *p* = 2.9 × 10^−09^, nested ANOVA (mouse nested within session) F = 4.6, *p* = 6.7 × 10^−11^, S3 F = 211, *p* = 1.7 × 10^−47^, nested ANOVA (mouse nested within session) F = 218.9, *p* = 5 × 10^−49^, and S5 F = 111.4, *p* = 6.4 × 10^−26^, nested ANOVA (mouse nested within session) F = 99.2, *p* = 2.8 × 10^−23^) and PV cell (2-way ANOVA, condition S1 F = 8.4, *p* = 0.003, nested ANOVA (mouse nested within session) F = 8.2, *p* = 0.0042, S3 F = 50.9, *p* = 1.8 × 10^−12^, nested ANOVA (mouse nested within session) F = 67.1, *p* = 8.1 × 10^−16^, and S5 F = 37.6, *p* = 1.2 × 10^−09^, nested ANOVA (mouse nested within session) F = 42.6, *p* = 1 × 10^−10^). While it increased for SST cells (2-way ANOVA, condition S1 F = 44, *p* = 3.7 × 10^−11^, nested ANOVA (mouse nested within session) F = 31.1, *p* = 2.5 × 10^−08^, S3 F = 61.2, *p* = 6.6 × 10^−15^, nested ANOVA (mouse nested within session) F = 56.6, *p* = 6.4 × 10^−14^, and S5 F = 159, *p* = 9.6 × 10^−36^, nested ANOVA (mouse nested within session) F = 129.2, *p* = 1.8 × 10^−29^). Values represent mean ± SE. (b) Change in activity per tracked cell in response to a 50 dB noise is defined as the slope of a linear fit of the mean activity of the baseline days and activity on days 1, 3, and 5 of stress (examples of the linear fit on the right). We found a negative slope in most PPY and PV cells, indicating a decrease in activity as the stress becomes chronic (*t* test for PPY cells *p* = 9.8 × 10^−14^, PV cells *p* = 0.006) and a positive slope for SST cells indicating an increase in activity with successive applications of the stressor (*t* test for SST cells *p* = 9.5 × 10^−06^). (c) Comparison of mean neural contrast between the pre-sound and post-sound windows for tracked PPY and SST cells in baseline and different repetitive stress sessions. The neural contrast was calculated as (post sound activity—pre sound activity)/(post sound activity + pre sound activity)*100 per cell. The difference in activity between the pre-sound and post-sound periods decreased for PPy cells (2-way ANOVA, condition S1 F = 18.9, *p* = 1.3 × 10^−05^, nested ANOVA (mouse nested within session) F = 15.3, *p* = 9 × 10^−05^, S3 F = 71.7, *p* = 2.8 × 10^−17^, nested ANOVA (mouse nested within session) F = 52.5, *p* = 4.6 × 10^−13^, S5 F = 56.7, *p* = 5.4 × 10^−14^, nested ANOVA (mouse nested within session) F = 63.5, *p* = 1.7 × 10^−15^) and increased for SST cells as the stressor became chronic (2-way ANOVA, condition S1 F = 42.4, *p* = 8.2 × 10^−11^, nested ANOVA (mouse nested within session) F = 4 3.2, *p* = 5.6 × 10^−11^, S3 F = 43.7, *p* = 4.3 × 10^−11^, nested ANOVA (mouse nested within session) F = 47.68, *p* = 5.9 × 10^−12^, S5 F = 127.1, *p* = 4.9 × 10^−29^, nested ANOVA (mouse nested within session) F = 126.2, *p* = 7.9 × 10^−29^). This was especially apparent for mid-sound intensities. Values represent mean ± SE. Source data for this figure can be found at: https://www.ebi.ac.uk/biostudies/studies/S-BSST1689754.

A continuous or repetitive state, such as repetitive stress, is expected to have a gradual and accumulating effect. To test whether this is the case and whether the change in noise-evoked activity develops over time, we examined the evolving cortical responses to noise as stress was repeated. Inspection of tracked single-cell activity per day revealed a striking decrease in sound-evoked activity for PPy (Fig 6A, top) and PV cells (Fig 6A, middle) and an increase in activity for SST cells (Fig 6A, bottom) as the stressor persisted over time. On the initial day of stress, the change in activity was relatively modest for all cell types, but it progressively increased by the third and fifth days. We found the same gradual and developing effect on the cortical response to the pure tone (S4A Fig). On the initial day of stress, there was no significant reduction in activity, but as the stressor became chronic, the reduction increased (S4A Fig, nested 2-way ANOVA S1 F = 2.8, *p* = 0.09, S5 F = 32.7, *p* = 1.3 × 10^−08^).

To quantify the gradual effect of stress on moderate sound intensities evoked activity, we examined the changes in activity per tracked cell across days in response to a 50 dB noise as the stressor was repeated. We used the slope of a linear fit of the mean activity of the baseline days and activity on days 1, 3, and 5 of stress as a measure of the effect of stress across days (Fig 6B, right). We found that most PPy and PV-tracked cells decreased their noise-evoked activity while SST cells increased their activity as the stress became chronic (Fig 6B, left, *t* test *p* < 0.05 for all cell types). We observed the same phenomenon in the modulation of neural contrast (Fig 6C) and in the pre- and post-sound deconvolved spike activity across different sound levels (S5 and S6 Figs). Additionally, this pattern was evident when quantifying changes in activity at other mid-intensities but not lower intensities (S4B Fig). These findings underscore the progressive nature of stress-induced changes in sound processing, indicating a cumulative effect that seems to be contingent upon sound intensity.

### Alterations in sound processing could have significant implications for perception

**Fig 7:**
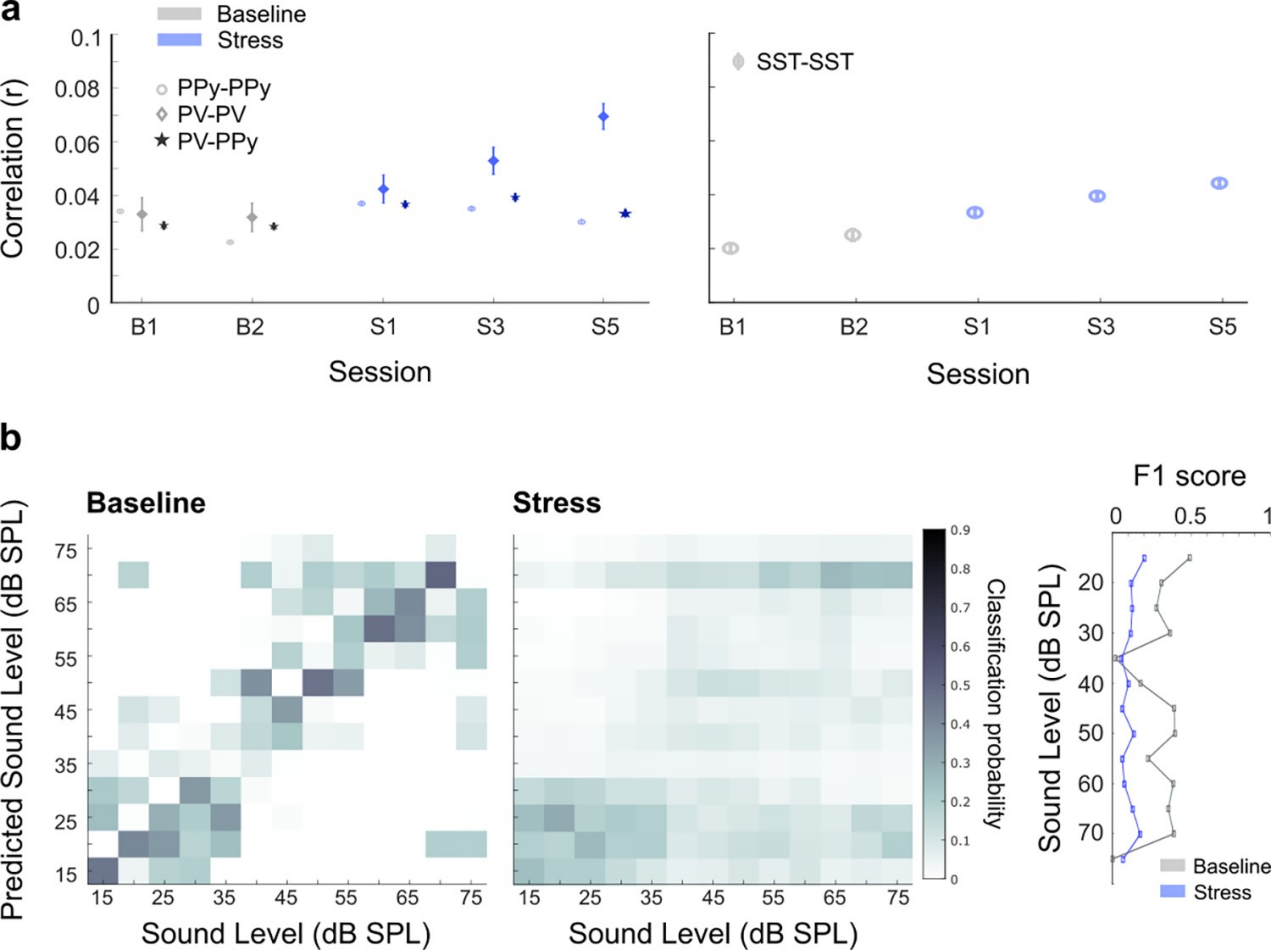
Alterations in sound processing could have significant implications for perception. (a) Left: Noise correlations between the normalized activity of pairs of PPy cells, PV cells, and a combination of both in the 500 ms surrounding the noise stimulus presentation at 50 dB SPL across the different sessions (mean ± SE). We found an increase in noise correlations for all pair types as the stressor was repeated (2-way ANOVA cell type:session F = 11.1, *p* = 7.8 × 10^−16^). Right: Same for SST cells (1-way ANOVA F = 39.5, *p* = 4.4 × 10^−33^). (b) Confusion matrices depict decoding accuracy for sound level based on population activity. Decoding was performed on 1,000 randomly drawn samples of 400 PPy units. Although the population activity in the ACtx could reliably decode sound intensity, this ability was impaired during repeated stress (all stress sessions were included in the analysis). Right, F1 scores, a measure of precision and recall, deteriorate with repeated stress exposure (mean ± SE). F1 scores were calculated as: (2*TP)/((2*TP) + FP + FN). TP—true positives, FP- false positives and FN—false negatives. Source data for this figure can be found at: https://www.ebi.ac.uk/biostudies/studies/S-BSST1689754.

If repetitive stress modulates the relationship between pre- and post- sound activity, it is reasonable to expect these changes to impact the functional connectivity and information flow within the network. To probe this possibility, we calculated noise correlations as a measure of trial-to-trial co-variability of responses, providing an estimate of mutual connectivity and shared inputs between and within cell classes [51]. Higher noise correlations during stimulus presentation limit the information capacity of a neural population and disrupt perception [45,52–54]. We compared the noise correlations between pairs of PPy cells, PV cells, and a combination of both in the 500 ms surrounding the noise stimulus presentation at 50 dB SPL. We observed an increase in noise correlations across all cell pair types (Fig 7A, left, 2-way ANOVA cell type:session F = 11.1, *p* = 7.8 × 10^−16^), potentially driven by heightened cortical activity resulting from repetitive stress. We observed the same increase in noise correlations for other mid-intensity sound responses (S7 Fig). The increase in noise correlations was minor on the first day of stress but increased as the stressor became chronic (Fig 7A, left). We repeated this analysis for SST cells and also found an increase in noise correlations that grew as the stressor was repeated (Fig 7A, right, 1-way ANOVA F = 39.5, *p* = 4.4 × 10^−33^).

Reduced proportional activity during sound presentation, combined with elevated noise correlations, may have significant implications for sound perception, as decreased sensitivity could hinder the accurate discrimination of changes in sound intensity. To explore whether these alterations in cortical activity lead to perceptual deficits, we employed a PSTH-based classifier to decode sound intensity from single-trial ensemble activity in the ACtx [55,56] (see Methods). Confusion matrices depict the mean predicted stimulus intensity based on ACtx activity versus the actual sound intensity played, where correct classification aligns with the diagonal (Fig 7B). While ACtx ensemble activity reasonably predicted stimulus intensity, classification accuracy was severely disrupted under repeated stress (Fig 7B, left versus middle). Often, sounds were misclassified as lower than their true intensity (Fig 7B, middle and right). This misclassification, coupled with the observed reduction in sound-evoked activity and elevated noise correlations, suggests that repetitive stress-induced changes in cortical activity may impair loudness perception in mice.

### Repetitive stress alters loudness perception

**Fig 8:**
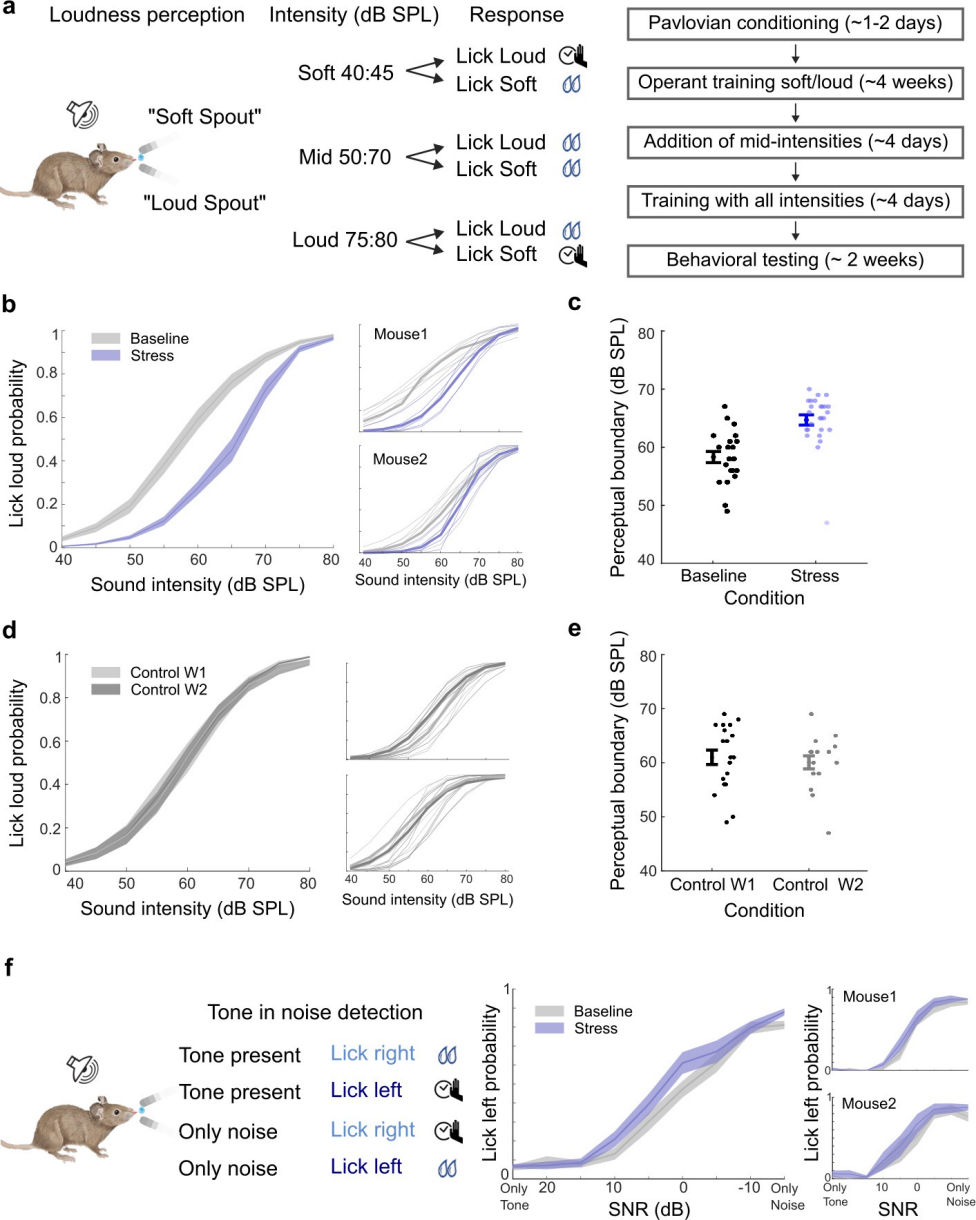
Repetitive stress modulates loudness perception. (a) Left: The schematic depicts the design of the head-fixed 2AFC loudness perception task. Mice were trained to categorize a white noise stimulus as either “soft” or “loud.” During testing, mice were required to correctly categorize 40–45 dB SPL (“soft”) and 75–80 dB SPL (“loud”) to receive a reward but were allowed to choose either spout for mid-level intensities ranging from 50- to 70-dB SPL. Right: an outline of the training timeline for this task (see Methods for more details). (b) Left: Mean lick loud probability in baseline (gray) and repeated stress (purple). During repeated stress, there was a reduced loudness reporting across moderate intensities (*N* = 6, mean ± SE, 2-way ANOVA condition F = 121.9, *p* = 5.9 × 10^−25^, intensities F = 347.2, *p* = 2.7 × 10^−175^, interaction F = 9.11, *p* = 1.5 × 10^−11^). There was no significant change in trained intensities 40–45- and 75–80-dB SPL (post hoc, *p* > 0.05 Bonferroni corrected). Right: exemplary behavior of 2 mice in baseline and repeated stress conditions. (c) Perceptual boundaries in different conditions. We define the perceptual boundary as the intensity where the psychometric fit to the choice function, crosses PLoud = 0.5. Mice exhibited increased perceptual boundaries during repeated stress, indicating a decreased loudness perception (*t* test, *p* = 1.2 × 10^−05^, mean ± SE). (d) Control animals (*n* = 3) underwent an identical learning process without experiencing stress. There was no change in loudness perception (2-way ANOVA condition F = 0.21, *p* = 0.64, intensities F = 190.8, *p* = 6.7 × 10^−119^, interaction F = 0.08, *p* = 0.9, mean ± SE). (e) In control animals, there was no change in perceptual boundary (mean ± SE, *t* test *p* = 0.6). (f) Left: Schematics depict the design of head-fixed 2AFC tone in noise detection task. Middle: There was no improvement in tone in noise detection during repeated stress (2-way ANOVA condition F = 0.9, *p* = 0.51, mean ± SE). Right: exemplary behavior of 2 mice. Source data for this figure can be found at: https://www.ebi.ac.uk/biostudies/studies/S-BSST1689754.

To test this prediction, we trained a group of mice in a two-alternative forced-choice operant loudness perception task [57], and tested their performance during baseline and stressful states using similar stimulus parameters as in the imaging experiments (Fig 8A, *N* = 6). As in the previous experiments, repetitive stress was induced by daily restraint. Mice were rewarded with sweetened water for licking the “soft spout” shortly following the presentation of low-intensity white noise (40 to 45 dB SPL), while the “loud spout” was associated with higher-intensity white noise (75 to 80 dB SPL). Their behavioral choice was measured over a 40 to 80 dB SPL range, with mice receiving conditional rewards for selections within the low and high ranges they trained on. Notably, mice were rewarded regardless of the spout choice for noise within the mid-intensity range (50 to 70 dB SPL, see Fig 8A). This reward structure ensured that loudness perception was exclusively governed by sound intensity, without influencing how mice responded to the sounds within the intermediate intensity range [57].

After the mice mastered the task, we assessed their performance for a week at baseline and another week in a stressful state. During baseline, mice accurately reported sounds at the low and high ends of the continuum (Fig 8B, gray) and alternately reported intermediate sound intensities as loud or soft (e.g., 55 dB SPL), yielding a choice probability curve when averaged across trials (Fig 8B, gray). Loudness reporting across behavioral sessions was highly reliable across all mice tested, with a perceptual boundary (calculated as the 50% choice probability of choosing loud) consistently occurring at approximately 60 dB SPL [57] (Fig 8C, black).

Despite undergoing repetitive stress, the same mice continued to perform the behavioral task, exhibiting appropriately timed lick behaviors. Their behavioral choices remained consistent with task demands, with correct reporting of the trained intensities at 40 to 45 and 75 to 80 dB SPL (Fig 8B, purple). However, for intermediate tone intensities (50 to 70 dB SPL), sounds previously categorized as loud were more frequently reported as soft under repetitive stress (Fig 8B, purple, 2-way ANOVA condition F = 5.9, *p* = 5.9 × 10^−25^, intensities F = 347.2, *p* = 2.7 × 10^−175^, interaction F = 9.11, *p* = 1.5 × 10^−11^). To quantify this shift in loudness perception, we calculated the perceptual boundary, which showed a significant increase during repetitive stress compared to baseline sessions (Fig 8C, *t* test, *p* = 1.2 × 10^−05^).

To determine whether this reduction in loudness perception resulted from the stressful state or the additional week of training, we trained a second group of mice (*N* = 3) following the same procedures but without experiencing daily restraint stress. These mice exhibited minimal changes in task performance (Fig 8D, 2-way ANOVA condition F = 0.9, *p* = 0.51), or perceptual boundary (Fig 8D, right, *t* test, *p* = 0.6). This suggests that the reduction in loudness perception was likely due to repetitive stress and not the extra week of training.

Chronic stress could affect all aspects of perception uniformly or selectively modulate certain aspects and not others. To test if the change observed is specific to loudness perception, we trained an additional group of mice in a tone-in-noise detection task. Since we found a decrease in activity in response to both noise and pure tones during chronic stress in our imaging experiments (Figs 2A and 4A), we predicted that there would be no improvement in tone in noise detection. This new group of animals was trained to lick to one side when they heard an 8 kHz tone and to the other side when there was no tone present (Fig 8F, *N* = 4). The mice were tested at different SNRs, for a week in baseline and another week under repeated stress. During baseline, mice accurately reported the presence or absence of 8 kHz tone, with better performance at higher SNRs (Fig 8F). Under chronic stress, mice continued participating in the task and displayed appropriately timed responses, and their performance neither improved nor deteriorated (Fig 8F, 2-way ANOVA condition F = 0.9, *p* = 0.51). Collectively, these results suggest that stressful states can influence perception, but not all aspects of perception are equally modulated.

## Discussion

Our findings contribute to a growing body of literature elucidating the impact of internal states on brain-wide dynamics [58–60]. Through the integration of changes in the animal’s internal state with longitudinal measurements of activity in the auditory cortex and auditory-guided behaviors, we revealed a possible mechanism via which repetitive stress can modulate sensory processing and the perception of neutral sounds in an intensity-dependent manner. Furthermore, our results illustrate that this effect progressively develops as the stressor becomes chronic.

Prior research has primarily explored how stress influences perception concerning stimuli with preexisting positive or negative connotations. For instance, acute stress has been linked to analgesic effects, while chronic stress has been associated with heightened sensitivity to pain [14,15]. The influence of stress extends to odor hedonics as well, impacting the attraction to odors that are initially perceived as either pleasant or unpleasant [13,16,17]. Our study goes further and demonstrates that the effects of repetitive stress extend beyond stimuli with inherent emotional associations. Repetitive stress also influences neutral stimuli encountered in everyday life, those lacking predetermined positive or negative connotations without affecting the periphery. Moreover, we show that repetitive stress significantly alters sensory perception, modulating loudness perception.

Acute stress often triggers a rapid increase in neuronal excitability in regions such as the hypothalamus [61], hippocampus, and the basolateral amygdala [62,63]. This surge in activity is mediated by the rapid actions of stress mediators, like corticosterone, on neuronal circuits [3]. In contrast, chronic or repeated stress can produce more complex and sometimes opposing effects on neuronal excitability. While certain brain regions show heightened excitability, others experience reduced excitability or impaired modulation of neuronal activity [3,64]. Consistent with this pattern, we observed a general increase in spontaneous activity of PPy cells in the auditory cortex during repeated stress. However, the proportional increase in sound-evoked activity was proportionally smaller, resulting in reduced neural contrast. This reduced contrast between spontaneous and sound-evoked activity may serve to decrease the salience of sound-related information transmitted to downstream areas.

Altogether, this diminished neural contrast may reflect an adaptive mechanism under repeated stress, contributing to the moderation of this excitable state. By dampening its response to auditory stimuli, the auditory cortex could be simplifying its already elevated processing load during stress. This adaptive strategy may facilitate conserving cognitive resources and enhancing their efficient allocation, similar to the shift toward habitual responses observed during chronic stress periods [65]. Furthermore, repetitive stress may contribute to heightened arousal and vigilance, prompting a selective focus on stimuli perceived as more immediately relevant or threatening. Consequently, auditory responses could be subdued in favor of other sensory modalities. Previous research has demonstrated that visual or tactile stimuli can modulate acoustically driven activity in the auditory cortex, often by suppressing responses to sound in both awake and anesthetized animals [66–68]. Hence, suppressing sound-evoked activity in cortical neurons may serve as a mechanism to prioritize the processing of tactile or visual cues from nearby objects requiring urgent attention. Similarly, locomotion can lead to a decrease in neural firing rates and sensory responses in the auditory cortex [69]. Therefore, reducing activity during repetitive stress could represent a strategy to streamline cognitive processing and optimize the allocation of attentional resources during a heightened state.

Interestingly, during all our experiments we observed a more pronounced reduction in sound-evoked activity at moderate sound intensities, with responses to high-intensity sounds returning to near baseline values. This observation bears resemblance to the varying impact of chronic stress on pain sensitivity, contingent upon the stimulus level. While chronic stress may heighten pain sensitivity in rats exposed to low-intensity innocuous stimuli, their response to high-intensity noxious stimuli remains comparable to unstressed controls [15], suggesting an inherent withdrawal response regardless of stress levels. Similarly, our study indicates that after a week of repetitive stress, significant changes may not be discernible in response to high-intensity sounds, as these intense stimuli tend to provoke a response regardless of the subject’s internal state. Viewed through the lens of adaptive mechanisms, the auditory system may down-regulate sensitivity to moderate-intensity stimuli, typically representing routine or non-threatening events, to conserve energy and attentional resources. Conversely, responses to high-intensity sounds, often indicative of imminent danger or important events, may increase or remain relatively unchanged, as heightened sensitivity to such signals is crucial for survival and immediate threat detection.

A recent study examined the long-term effects of 4 weeks of corticosterone treatment on frequency-specific activity in the auditory cortex [70]. One week after the treatment ended, they observed an increase in local field potentials in response to an 8 kHz tone burst at 70 dB SPL but not for 12 or 16 kHz tones. Similarly, they observed an increased activity for 8 and 12 kHz tone bursts at 90 dB SPL, but not for 16 kHz. Notably, unlike our 1-week mild restraint stress protocol, this extended glucocorticoid treatment led to higher glucocorticoid receptor expression in the auditory cortex. This raises intriguing questions about whether sound processing and perception changes are influenced by stress severity and whether the stress induces glucocorticoid receptor expression changes. It also raises the question of how long the effects of repetitive stress may persist after the stressor is discontinued.

The literature on GABAergic dysfunction in stress and affective disorders is inconclusive and, at times, contradictory. While many studies report decreased GABAergic function, some have found evidence for increased inhibition [39,43,50,71–74]. We sought to determine whether the decline in activity we observed in PPy cells could be attributed to an increase in the activity of inhibitory PV cells. We found this was not the case, as PV cells also exhibited reduced activity (Fig 2), consistent with the susceptibility of parvalbumin neurons to chronic stress in the prefrontal cortex [50] and hippocampus [40]. One possibility is that the balance between inhibition and excitation remains stable, leading to a reduction in overall sound-evoked activity in the ACtx. Another potential explanation is that a different type of inhibitory cell could be driving the decrease in PPy sound-evoked activity. Research on the impact of chronic stress on inhibitory neurons has shown an increase in the expression of somatostatin in the dorsal hippocampus of male rats subjected to 7 weeks of mild stress [40]. Additionally, the structure of somatostatin-expressing cells was found to be affected by chronic stress, with an increase in the complexity of their dendritic arbor in adult male mice [50]. These findings raise the possibility that changes in the activity of somatostatin-expressing inhibitory cells could play a role in the reduction in activity we observed in both PPy and PV cells.

To discern between these possibilities, we conducted a new set of experiments where we tracked the activity of SST cells during repeated stress. We found that while SST cells also showed an increase in pre-sound activity, unlike PPy and PV cells, SST cells showed an increase in tone-evoked activity. This suggests that the increase in sensory-evoked activity in SST cells may be driving the reduction in sound-evoked activity in PPy and PV cells. The central role of SST cells in modulating sound-evoked activity is not limited to repetitive stress but seems to be a general mechanism across sensory cortices [75–77]. This is likely due to their broad input connectivity [76,78] and the absence of self-inhibition [79]. These results pave the way for manipulation experiments to test whether inhibiting SST activity during repetitive stress reduces its impact on sound-evoked activity in PPy cells.

Our work also reveals that the impact of stress on sensory processing evolves gradually as the stressor persists over time (Fig 6). Our work shows that the impact of stress on auditory processing undergoes changes when transitioning from an acute to a persistent state, underscoring the dynamic and evolving nature of this relationship. Work on the effect of acute stress on sound processing found that the neural activity in the auditory cortex was significantly modulated by restraint stress but that the changes completely disappeared 30 min after the animal was released [80]. Similarly, we observed minimal effects of stress on the first day; however, these effects grew as the stressor became chronic. The gradual impact of repeated stress on sound processing may be attributed to a range of physiological and psychological processes that unfold as stress persists, including hormonal shifts, alterations in gene expression, and changes in neuronal structure [3]. Some of these processes may facilitate adaptation to a new physiological state, potentially explaining the greater recovery of responses to high noise intensities compared to intermediate intensities observed after 5 days of stress. These findings hold significance when considering strategies for learning and decision-making, 2 domains significantly affected by prolonged stress exposure. Previous research has suggested that chronic stress can lead to more rigid stimulus-response learning patterns [81] and habitual decision-making strategies [65]. The alterations in sensory processing and perception identified in our study may play a role in shaping these learning and decision-making approaches. For instance, the reduction in sound-evoked activity could influence the prioritization of information for learning or affect the encoding and retention of auditory information, potentially impacting decision-making processes.

The observed alterations in sound processing could have significant implications for perception. Our findings, including increased noise correlations, and a decline in the neural decoder’s ability to categorize sound intensities (Fig 7), suggest a potential modulation of loudness perception, particularly concerning moderate sound intensities, during chronic stress. To test this hypothesis, we trained mice in a 2-AFC loudness perception task [57], revealing that mid-intensity sounds perceived as loud during baseline measurements were more often reported as soft during chronic stress (Fig 8). The shift towards decreased loudness is consistent with our findings of reduced sound-evoked activity in mid-intensities in our imaging experiments (Fig 2).

Importantly, this behavioral task specifically assesses loudness perception, as either spout choice in the mid-intensities tested results in a water reward for the animal. Still, to address the possibility that chronic stress affects other behavioral features, we train an additional group of mice in a different auditory task—tone-in-noise detection. Here, we did not observe comparable alterations in performance (Fig 8F). These results, together with the consistent performance in the trained intensity levels required to obtain conditional water rewards within the loudness perception task, strongly suggest that the observed changes in loudness perception are not contingent upon chronic stress affecting learning or attention. The reduction in both tone-evoked and noise-evoked activity (Figs 2 and 4) may explain why there is neither improvement nor deterioration in tone-in-noise detection capabilities as the levels of both the target and the background-evoked activity are reduced. Another possibility could be that while some aspects of perception are subject to modulation by chronic stress, others may reach a plateau or threshold where further reduction or enhancement becomes challenging, regardless of modulation efforts.

Changes in perception, such as modulations in loudness perception, during stressful states may enhance our chances of evolutionary survival by prioritizing the processing of more urgent or more informative sensory stimuli. However, these alterations in perception could also become maladaptive, potentially exacerbating or even triggering mental and sensory disorders [82–85]. Given the importance of accurate sensory processing for higher cognitive functions, these progressive changes in auditory processing could potentially impact areas like attention, memory, and decision-making. Indeed, chronic or repetitive stress in modern contexts can elicit a range of physiological and psychological responses that may not always be beneficial for our overall well-being. Our results reveal a new possible mechanism through which repetitive stress may modulate behavior and underscore the importance of considering the impact of repetitive stress on sensory processing and perception, especially in discussions involving complex processes such as learning and stress-related disorders.

## Materials and methods

### Resource availability

The source data of this paper is collected in the following free access database: BioStudies, accession number S-BSST1689: https://www.ebi.ac.uk/biostudies/studies/S-BSST1689.

This study did not generate new unique reagents.

### Experimental model and subject details

All procedures were approved by the Ben-Gurion University Animal Care and Use Committee (approval No. IL-07-01-2022D). Data were collected from 73 adult mice (10 to 16 weeks postnatal, PV-Cre x Ai14, JAX stock no: 017320 and 024109, respectively and SST-IRES-Cre JAX stock no:13044). Twenty-two mice contributed to the different behavioral tasks, 14 to the imaging experiments, 6 to the histology, 3 to the ABRs, and 28 to the corticosterone measurements. Mice of both sexes were used for this study. Mice were maintained on a reverse 12 h light/12 h dark cycle and were provided with ad libitum access to food and water unless they were undergoing behavioral testing, in which case they had restricted access to water in the home cage.

### Restraint stress

Before the relevant imaging and behavioral sessions, the mice were placed in a 50 ml tube for 30 min to achieve mild stress. This was done once a day for a week. The imaging or behavioral session started directly after the restraint.

### Corticosterone measurement

Mice were randomly assigned to 2 groups for corticosterone measurement at different post-stress time points. Group 1 was measured at baseline, 30 min, and 80 min post-stress, while Group 2 was measured at baseline, 50 min, and 120 min post-stress. Animals were also randomly assigned to specific measurement days: 14 mice were tested on days 1 and 3, another 14 on day 5, and 13 on day 7. One animal was unable to provide a sufficient blood sample on day 7, resulting in a smaller group. Each mouse was measured at baseline and 2 distinct post-stress time points and days to provide balanced data. From the animals that completed day 7, we randomly selected 6 to continue in the experiment beyond the stress phase. Tail blood samples were collected to determine the corticosterone levels. Blood samples were temporarily placed in iced plastic tubes coated with heparin. All serum was prepared after every blood sample was centrifuged at 10,000 rpm for 10 min. The supernatant was collected, and plasma corticosterone concentration was measured using commercially available ELISA kits (Enzo ADI-900–097).

### Glucocorticoid receptor (GR) expression

Mice underwent 7 days of retrain stress (*n* = 3) or no stress (control *n* = 3). Then, all the mice were perfused (4% PFA) and their brains were removed. The brains were embedded in parafilm, coronal sections were obtained from the auditory cortex (selected to match the Allen brain atlas) and stained IHC with antibody for Glucocorticoid receptor (Glucocorticoid receptor Polyclonal antibody 24050-1-AP Proteintech). Quantification of GR expression was performed using Image J software. A threshold was set to separate positive GC stains from the background and the percentage of the area stained was calculated. GR expression was evaluated in 3 sections for each stressed and control mouse.

### Auditory brainstem responses

Mice were anesthetized with ketamine (120 mg/kg) and xylazine (12 mg/kg) and placed on a homeothermic heating blanket during testing. Half the initial ketamine dose was given as a booster when required. ABR stimuli were 5 ms tone pips at 16 kHz with a 0.5 ms rise-fall time delivered at 30 Hz. Intensity was incremented in 5 dB steps, from 20 to 80 dB SPL. ABR threshold was defined as the lowest stimulus level at which a repeatable waveform could be identified. Wave 1 amplitudes were calculated for the different sound intensities. ABRs were measured in the same mice at baseline and after 7 days of daily restraint stress.

### Open field

Each mouse was placed individually in the open field arena, and its movement was captured using an Ezviv camera placed approximately 1 meter above the center of a white lusterless Perspex box (60 cm [W] × 60 cm [L] × 60 cm [H]), divided into a 25% central zone and a 75% peripheral zone. Data was analyzed using the Ethovision tracking system (Etho-Vision XT 14; Noldus Information Technology, the Netherlands). We tested mice on day 1 and again on day 7. Mice in the repeated stress group underwent restraint stress the whole week, even on days with no behavioral testing.

### Survival surgeries for awake, head-fixed imaging, and behavior experiments

Mice were anesthetized with isoflurane in oxygen (5% induction, 1.5% maintenance). The dorsal surface of the mice’s heads was trimmed and sterilized. ThermoStar homeothermic blanket monitoring system was used to maintain body temperature at 36.6°C (RWD). Lidocaine hydrochloride was administered subcutaneously to numb the scalp. The dorsal surface of the scalp was reduced using surgical scissors, and the periosteum was removed. The skull surface was prepped with an etchant (C&B metabond) and vetbond (3M) before affixing a custom stainless-steel headplate to the dorsal surface with dental cement (C&B metabond). At the conclusion of the headplate attachment and any additional procedures listed below, Buprenex (0.05 mg/kg) and meloxicam (0.1 mg/kg) were administered, and the animal was transferred to a warmed recovery chamber.

### Virus-mediated gene delivery

For mice used in the PPy and PV imaging experiments, 2 burr holes were made in the skull over the auditory cortex (1.75 to 2.25 mm rostral to the lambdoid suture) of PV-Cre x Ai14 mice. A precision injection system (Nanoject III) was used to inject 75 nL of AAV5.Syn.GCaMP6s.WPRE.SV40 in each burr hole 200 to 250 mm below the pial surface. Before starting the imaging sessions, we waited approximately 3 weeks of virus incubation. For SST and the second PV experiments, the same protocol was used while injecting AAV5-syn-FLEX-jGCaMP8f-WPRE to SST-IRES-Cre and PV-Cre mice.

### Two-photon calcium imaging

Three round glass coverslips (one 4 mm, two 3 mm, #1 thickness) were etched with piranha solution and bonded into a vertical stack using transparent, UV-cured adhesive. Headplate attachment, anesthesia and analgesia follow the procedure described above. A 3 mm craniotomy was made over the right ACtx using a scalpel and the coverslip stack was cemented into the craniotomy. An initial widefield epifluorescence imaging session was performed to visualize the tonotopic gradients of the auditory cortex and identify the position of A1 [86]. Two-photon excitation was provided by a Ti:Sapphire-pulsed laser tuned to 940 nm. Imaging was performed with a 163/0.8NA water-immersion objective (Nikon) from a 512 × 512 pixel field of view at 30 Hz with a Galvo-Resonant 8 kHz scanning microscope (Thorlabs). Scanning software was synchronized to the stimulus generation hardware using digital pulse trains. The microscope was rotated by 50 to 60 degrees off the vertical axis to obtain images from the lateral aspect of the mouse cortex while the animal was maintained in an upright head position. Imaging was performed in a light-tight, sound-attenuating chamber mounted on a floating table. Animals were monitored throughout the experiment to confirm all imaging was performed in the awake condition. Imaging was performed in layers L2/3, 200 to 250 mm below the pial surface. Fluorescence images were captured at 2× digital zoom, providing an imaging field of (0.42 × 0.42 mm). Raw calcium movies were processed using Suite2P [44], a publicly available two-photon calcium imaging analysis pipeline.

We imaged a total of 5,338 PPY cells, 529 PV cells, 1,246 SST cells, and 696 PV cells in the separate experiment. Then, cross-day image registration was done with the registers2p function in Suite 2P, using the green (GCaMP) channel and red (tdTomato) channel when possible. This approach allowed us to identify the same cells across days and follow these cells throughout the baseline and stress sessions. Using the cross-day analysis, we tracked cells along all the sessions—baseline and stress. In all the analyses, except in S2 Fig, only cells that were sound responsive, and were identified in at least 1 baseline session and 1 stress session were included in the analysis. This included 285 PPY cells, 31 PV cells, 121 SST cells, and 81 PV cells in the separate experiment.

ΔF/F was computed as follows: (F(t)–F0)/F0, where F(t) was the raw calcium signal and F0 was the mean baseline fluorescence prior to stimulus presentation across trials. Spike deconvolution was also performed in Suite2P [44], using the default method based on the OASIS algorithm [44,87,88].

### Auditory stimulus for imaging experiments

Auditory stimuli were generated with a 24-bit digital-to-analog converter (National Instruments model PXI-4461) using scripts programmed in MATLAB (MathWorks) and LabVIEW (National Instruments). Speakers were calibrated for their distance from the contralateral ear (left ear) of the mouse. For imaging experiments, we played every other day a 100 ms 12 kHz tone in a quiet environment (20 to 80 dB SPL with 10 dB SPL step), a 100 ms white noise generated from a Gaussian distribution at different sound intensities (15 to 75 dB SPL with 5 dB SPL step) and a 500 ms 4 to 20 kHz FM sweep at 30-, 50-, and 70-dB SPL. All the trials had a 3.5 duration. Each stimulus was repeated 30 times. This strategy was designed to keep imaging sessions brief and to avoid habituation and photobleaching caused by prolonged exposure to repetitive stimuli on consecutive days.

Two-alternative forced choice frequency discrimination and tone in noise detection tasks.

Mice were water-restricted before starting the behavioral training until reaching 85% to 80% of their original weight and were given supplemental water if they received less than 1 ml during a training session. Mice required approximately 3 to 4 weeks of behavioral shaping before they could perform the complete 2AFC task. For shaping, mice were first habituated to head-fixation and Pavlovian conditioned. In the Loudness perception task [57]: White noise at 40 and 45 dB SPL was presented with a small quantity (4 μl) of water on the “soft” spout while 75 and 80 dB SPL tones were paired with water on the “loud” spout (*Pavlovian conditioning step*). The assignment of loud and soft to the left and right spouts was randomly determined for each mouse. In the next step, mice needed to lick the correct spout (loud or soft) to get the water reward (*operant training soft/loud step*). Once mice met the criterion of >80% correct categorization, we started adding the mid intensities (50 to 70 dB SPL, *addition of mid-intensities step*) until they reached the full stimulus set, where tone intensity on each trial was randomly selected from a 40 to 80 dB SPL (5 dB step size). At this stage, mice were required to correctly categorize 40 to 45 dB SPL tones as “soft” and 75 to 80 dB SPL tones as “loud” by licking the appropriate spouts to receive water, but they were rewarded regardless of their spout choice for 50 to 70 dB SPL tones (*training with all intensities step*). Mice were acclimated to the full psychometric form of the task for approximately 4 days, and then behavioral testing began (*behavioral testing step*).

In the tone in noise detection task: The animals were trained to lick to one side when they heard an 8 kHz tone and to the other side when there was only background noise (i.e., no tone) present. The assignment of tone and “no tone” to the left and right spouts were randomly determined for each mouse. Once mice met the criterion of >80% correct categorization, they were advanced to the full stimulus set, where the tone was played with different intensities of background noise and the mice were tested at different SNRs (only tone, −10:5:20 dB SNR and only noise).

For both tasks, after the mice mastered the task, they were tested for 1 week in baseline, followed by 1 week of repeated stress. During stressful sessions, mice were restrained for 30 min before starting the behavioral task. Hits were defined as post-target licks occurring more than 0.15 s but less than 1.2 s following the onset of the target tone. The lick loud/left probability was calculated as the number of times the mouse licked loud/left proportional to the number of times the specific tone was presented. Psychometric functions were fitted using binary logistic regression, and the perceptual boundary was defined as the point at which the lick loud probability equaled or exceeded 50%. Control mice underwent the same training and testing but without repeated stress. For the behavioral analysis, we included the second day of stress and the subsequent days, as the behavioral results in the first day of stress might be the response to acute stress.

### Two-photon calcium imaging analysis

Imaging data was analyzed both as ΔF/F and deconvolved spikes. A cell was considered sound responsive if the mean z-score activity relative to the baseline activity during a 300 ms post-stimulus window was greater than a 1.2 std threshold in 2 consecutive sound intensities for at least 1 baseline session and 1 stress session. Sound-evoked activity rates were calculated as the mean activity during a 300 ms post-stimulus window.

Change in activity between baseline and stress states (Fig 2) was calculated as the difference between the mean response in the baseline sessions and stress sessions per cell. Neural contrast was calculated as (post-sound activity—pre-sound activity)/(post-sound activity + pre-sound activity)*100. The pre-sound window was defined as the average activity 300 ms before the sound, and the post-sound period as the average activity 300 ms after the sound’s onset.

Change in activity across days (Fig 6) was calculated by fitting a regression line across the mean 50 dB SPL noise-evoked activity in the baseline days and days 1, 3, and 5 of repeated stress. We calculated the slopes per cell using an r^2^ > 0.75 goodness of fit. Same analysis was done for 40, 60, and 70 dB SPL noise evoked activity in S4B Fig.

We quantified noise correlations as the Pearson’s correlation coefficient between normalized activity of PPy-PPy, PV-PV, and mixed PPy-PV and SST-SST pairs, per session with the “Corrcoef” function in MATLAB. Pair-wise noise correlations were calculated in a window of 500 ms following the white noise onset. If there was no activity at all through the entire window, the trial was discarded. From the entire noise correlations matrix, values were averaged over cell pairs and sessions.

The input to population-PSTH classifiers was a vector of the normalized deconvolved spike activity in the 300 ms post-stimulus period for a randomly drawn set of sound-responsive 400 PPy units. For a given sound level, the population activity on the held-out trial was compared to the mean population rates across all intensities in the training set. The single trial response was classified as the stimulus to which the Euclidean distance is the smallest [55,56]. The classification was repeated until each trial was used as the held-out test trial. The overall classifications were averaged across 1,000 random draws of PPy units. The precision and recall of the classifier was calculated using the F1 score defined as: (2*TP)/((2*TP) + FP + FN). TP—true positives, FP- false positives, and FN—false negatives.

### Statistical analysis

All statistical analyses were performed in MATLAB R2023a (Mathworks). Data shown in all analyses is the mean activity ± SE unless otherwise indicated. Post hoc pairwise comparisons were corrected for multiple comparisons using the Bonferroni correction. To conduct nested ANOVAs, we identified and matched the data corresponding to each mouse across repeated sessions to account for within-subject variability. A nested ANOVA was conducted using MATLAB’s Anovan function with a nested model. The dependent variable was cell activity, and the independent variables were session and mouse (nested within sessions). We specified the nested structure to account for inter-mouse variability and ensure that any session-level effects are not confounded by mouse-specific differences. This approach allowed us to test for significant effects of the dependent variables of interest—cell activity—across sessions.

## Supporting information

S1 Fig Repetitive restraint stress does not affect auditory brainstem responses or glucocorticoid receptor expression. (a) Left: Example of glucocorticoid receptor expression during a baseline session. Right: Repeated stress did not change the expression of glucocorticoid receptors (3 stress exposed and 3 control mice, 3 slides per mice, *t* test, *p* = 0.9, mean ± SE). (b) Mean ABR wave 1 amplitude and threshold at 16 kHz for 3 mice during baseline and after a week of daily restraint stress. There was no change in wave 1 amplitude (left, 2-way ANOVA condition F = 0.01, *p* = 0.8, condition:level interaction F = 1, *p* = 0.44) or ABR threshold (right, *t* test, *p* = 1, mean ± SE) during repeated stress. Source data for this figure can be found at: https://www.ebi.ac.uk/biostudies/studies/S-BSST1689754.
(TIFF)

S2 Fig Modulation of sound responsiveness during repetitive stress. (a) Percentage of sound-responsive cells in baseline and repeated stress conditions for PPy and PV cells (all imaged cells). There was a small and significant decrease in the percentage of responsive PPy cells (*n* = 10 sessions in baseline and 15 sessions during stress, *t* test, *p* = 0.02) and a nonsignificant decrease for PV cells (mean ± SE, *n* = 190 cells in baseline and 339 cells during stress, *t* test, *p* = 0.19). (b) Percentage of sound-responsive cells in baseline and repeated stress conditions for all SST cells (all imaged cells). There was a nonsignificant increase for SST cells (mean ± SE, *n* = 8 sessions in baseline and 12 sessions during stress, *t* test, *p* = 0.1). Source data for this figure can be found at: https://www.ebi.ac.uk/biostudies/studies/S-BSST1689754.
(TIFF)

S3 Fig Modulation of PV cells during repetitive stress. (a) Mean noise-evoked activity (deconvolved spikes) for different noise intensities in the baseline (gray) and repetitive stress (blue) for chronically tracked PV cells (*N* = 2 mice, *n* = 81 cells). The activity was averaged over a 300 ms period before (soft) and after the sound (dark colors). There was an increase in pre- and post-sound activity during repeated stress (mean ± SE, 3-way ANOVA, condition F = 576.1, *p* = 1 × 10^−122^, nested ANOVA (mouse nested within session) F = 566, *p* = 1.2 × 10^−120^). (b) Mean neural contrast between the pre-sound and post-sound windows for tracked PV cells in baseline and repetitive stress. The neural contrast was calculated as (post sound activity—pre sound activity)/(post sound activity + pre sound activity)*100 per cell. There was a decrease in neural contrast during repeated stress (mean ± SE, 2-way ANOVA, condition F = 97.4, *p* = 1 × 10^−222^, nested ANOVA (mouse nested within session) F = 98.1, *p* = 7.3 × 10^−23^). (c) The activity per PV cell was normalized (z-score) before calculating the mean noise-evoked activity. There was a decrease in sound-evoked activity during repeated stress (mean ± SE, 2-way ANOVA, condition F = 117.3, *p* = 5.9 × 10^−27^, nested ANOVA (mouse nested within session) F = 104.6, *p* = 3 × 10^−24^). Source data for this figure can be found at: https://www.ebi.ac.uk/biostudies/studies/S-BSST1689754.
(TIFF)

S4 Fig Repetitive stress induces a reduction in sound-evoked activity that develops as the stressor becomes chronic. (a) Change of tone-evoked activity over time. Comparison of tone-evoked activity after a day and 5 days of stress to baseline. On the initial day of stress, there was no significant reduction in activity, but as the stressor became chronic, the reduction increased (mean ± SE, 1-way ANOVA, condition S1 F = 1.9, *p* = 0.16, S5 F = 27.5, *p* = 1.7 × 10^−07^, nested ANOVA (mouse nested within session) condition S1 F = 2.8, *p* = 0.09, S5 F = 32.7, *p* = 1.3 × 10^−08^). (b) Change in activity across sessions per tracked cell in response to 40, 60, and 70 dB white noise. We found a negative slope in most cells especially for mid-intensities, indicating a decrease in activity as the stress becomes chronic (*t* test for 40 dB *p* = 0.36, for 60 dB *p* = 9.3 × 10^−12^, and for 70 dB *p* = 0.00750). Source data for this figure can be found at: https://www.ebi.ac.uk/biostudies/studies/S-BSST1689754.
(TIFF)

S5 Fig Modulation of PPy pre- and post-sound activity during repetitive stress. Deconvolved spikes in the pre- and post-sound periods (soft and dark colors accordingly) for PPy cells at different sound intensities in the baseline (gray/black) and stress (light blue/blue) sessions. The pre-sound window was defined as the average activity 300 ms before the sound, and the post-sound period as the average activity 300 ms after the sound’s onset. Values indicate mean ± SE. Source data for this figure can be found at: https://www.ebi.ac.uk/biostudies/studies/S-BSST1689754.
(TIFF)

S6 Fig Modulation of SST pre- and post-sound activity during repetitive stress. Same as S4 Fig but for SST cells. Deconvolved spikes in the pre- and post-sound periods (soft and dark colors accordingly) for SST cells at different sound intensities in the baseline (gray/black) and stress (light blue/blue) sessions. The pre-sound window was defined as the average activity 300 ms before the sound, and the post-sound period as the average activity 300 ms after the sound’s onset. Values indicate mean ± SE. Source data for this figure can be found at: https://www.ebi.ac.uk/biostudies/studies/S-BSST1689754.
(TIFF)

S7 Fig Increased noise correlations in mid-sound intensities during stress. (a) Noise correlations between the normalized activity of pairs of PPy cells, PV cells, and a combination of both in the 500 ms surrounding the noise stimulus presentation at 55 and 60 dB SPL across the different sessions. We found an increase in noise correlations for all types of pairs (2-way ANOVA cell type, session F = 9.5 and 12.2, *p* = 2.4 × 10^−13^ and 1.3 × 10^−17^, 55 and 60 dB, accordingly). The increase in noise correlations was particularly striking for PV-PV pairs; it was minor the first day of stress but increased as the stressor became chronic (post hoc B1 vs. S3 *p* = 0.004, B1 vs. S5 *p* = 2.2 × 10^−07^, B2 vs. S3 *p* = 3.3 × 10^−04^, B2 vs. S5 *p* = 3.3 × 10^−09^, all Bonferroni corrected). Source data for this figure can be found at: https://www.ebi.ac.uk/biostudies/studies/S-BSST168975.
(TIFF)
