# Supplementary figures and images for: Repeated stress gradually impairs auditory processing and perception
Source: PLoS Biol. 2025 Feb 11;23(2):e3003012. doi: 10.1371/journal.pbio.3003012 (PMC11813133; doi:10.1371/journal.pbio.3003012)

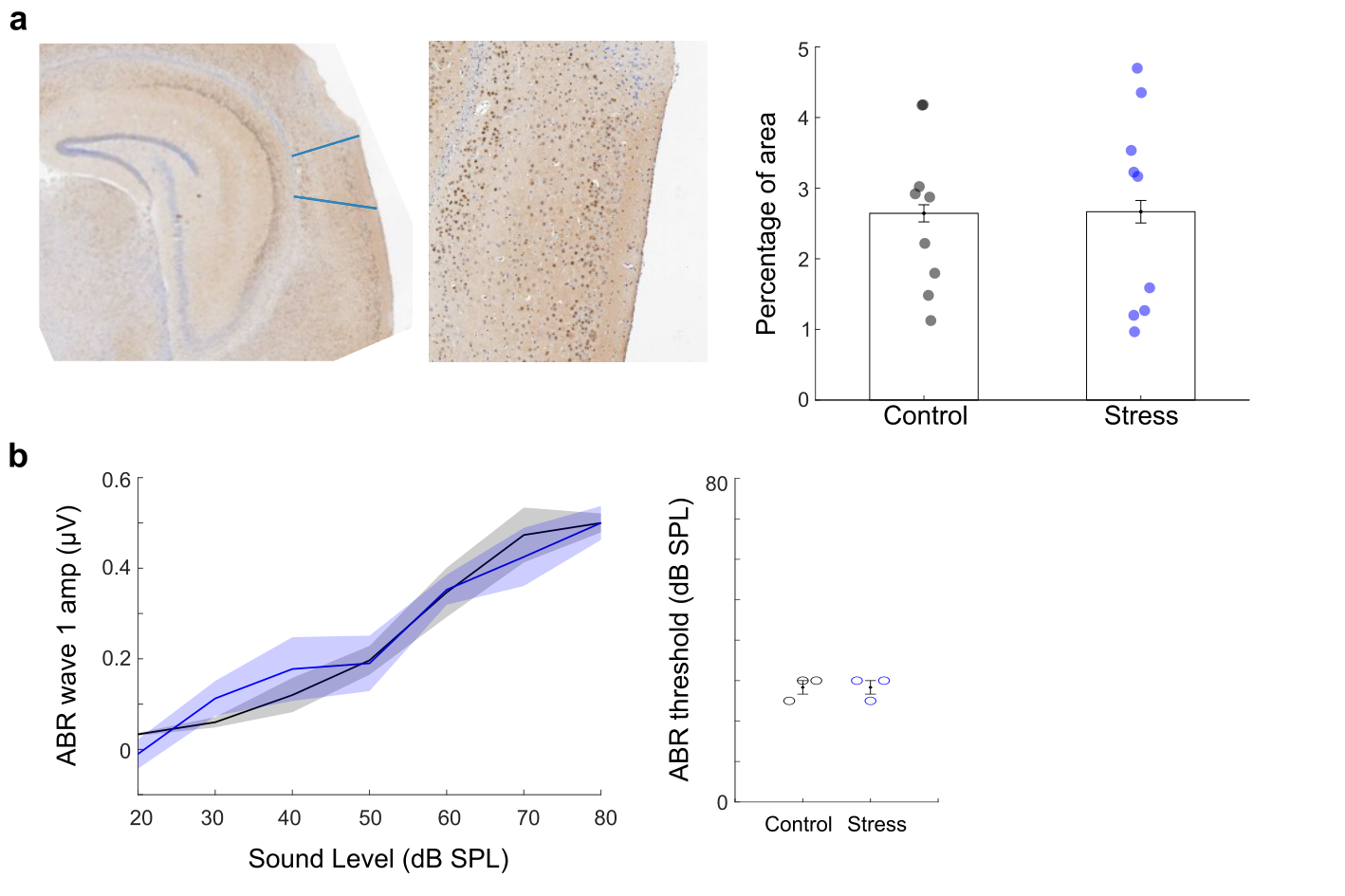

Supplement: S1 Fig — (a) Left: Example of glucocorticoid receptor expression during a baseline session. Right: Repeated stress did not change the expression of glucocorticoid receptors (3 stress exposed and 3 control mice, 3 slides per mice, t test, p = 0.9, mean ± SE). (b) Mean ABR wave 1 amplitude and threshold at 16 kHz for 3 mice during baseline and after a week of daily restraint stress. There was no change in wave 1 amplitude (left, 2-way ANOVA condition F = 0.01, p = 0.8, condition:level interaction F = 1, p = 0.44) or ABR threshold (right, t test, p = 1, mean ± SE) during repeated stress. Source data for this figure can be found at: https://www.ebi.ac.uk/biostudies/studies/S-BSST1689754. (TIFF) [file pbio.3003012.s001.tiff]

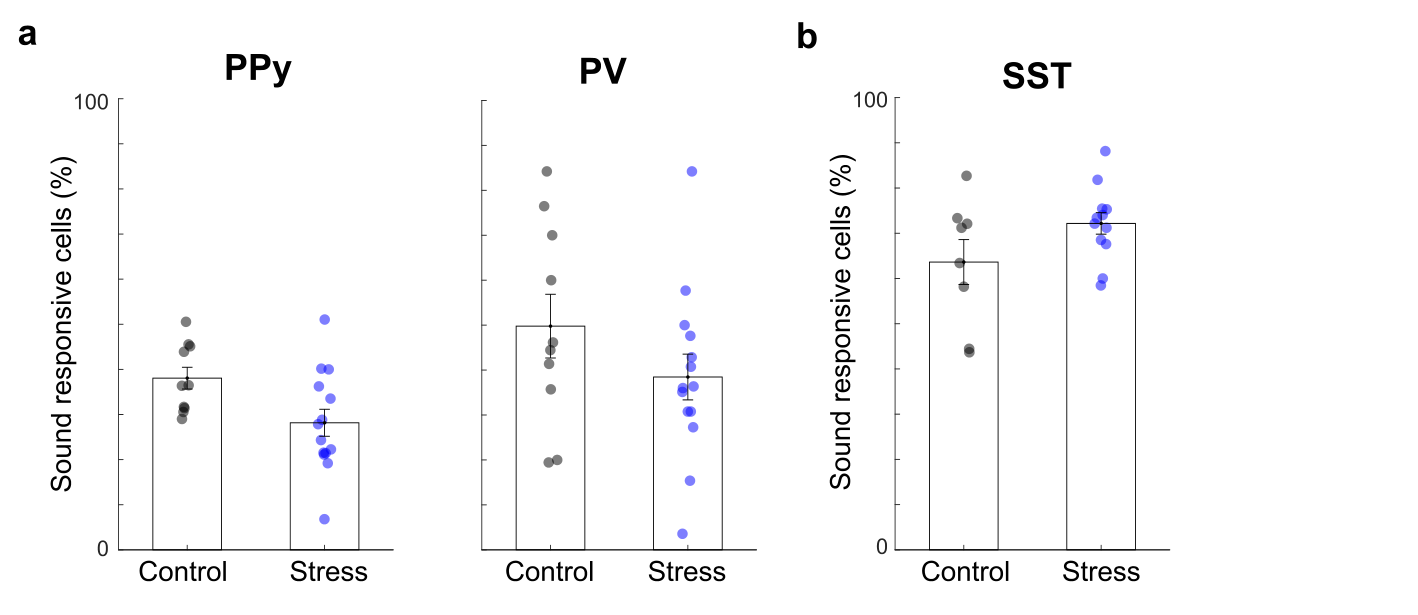

Supplement: S2 Fig — (a) Percentage of sound-responsive cells in baseline and repeated stress conditions for PPy and PV cells (all imaged cells). There was a small and significant decrease in the percentage of responsive PPy cells (n = 10 sessions in baseline and 15 sessions during stress, t test, p = 0.02) and a nonsignificant decrease for PV cells (mean ± SE, n = 190 cells in baseline and 339 cells during stress, t test, p = 0.19). (b) Percentage of sound-responsive cells in baseline and repeated stress conditions for all SST cells (all imaged cells). There was a nonsignificant increase for SST cells (mean ± SE, n = 8 sessions in baseline and 12 sessions during stress, t test, p = 0.1). Source data for this figure can be found at: https://www.ebi.ac.uk/biostudies/studies/S-BSST1689754. (TIFF) [file pbio.3003012.s002.tiff]

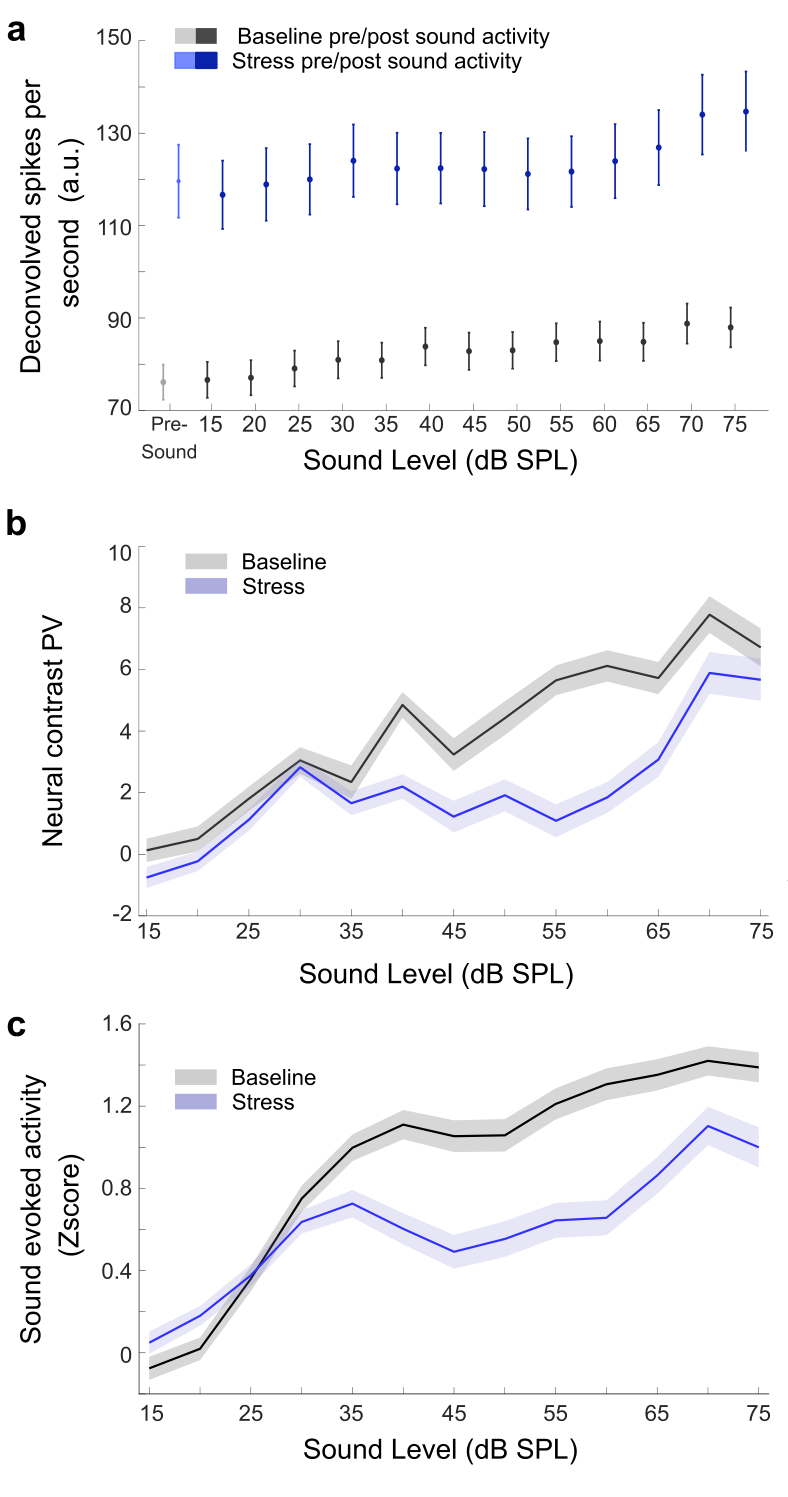

Supplement: S3 Fig — (a) Mean noise-evoked activity (deconvolved spikes) for different noise intensities in the baseline (gray) and repetitive stress (blue) for chronically tracked PV cells (N = 2 mice, n = 81 cells). The activity was averaged over a 300 ms period before (soft) and after the sound (dark colors). There was an increase in pre- and post-sound activity during repeated stress (mean ± SE, 3-way ANOVA, condition F = 576.1, p = 1 × 10−122, nested ANOVA (mouse nested within session) F = 566, p = 1.2 × 10−120). (b) Mean neural contrast between the pre-sound and post-sound windows for tracked PV cells in baseline and repetitive stress. The neural contrast was calculated as (post sound activity—pre sound activity)/(post sound activity + pre sound activity)*100 per cell. There was a decrease in neural contrast during repeated stress (mean ± SE, 2-way ANOVA, condition F = 97.4, p = 1 × 10−222, nested ANOVA (mouse nested within session) F = 98.1, p = 7.3 × 10^−23^). (c) The activity per PV cell was normalized (z-score) before calculating the mean noise-evoked activity. There was a decrease in sound-evoked activity during repeated stress (mean ± SE, 2-way ANOVA, condition F = 117.3, p = 5.9 × 10^−27^, nested ANOVA (mouse nested within session) F = 104.6, p = 3 × 10^−24^). Source data for this figure can be found at: https://www.ebi.ac.uk/biostudies/studies/S-BSST1689754. (TIFF) [file pbio.3003012.s003.tiff]

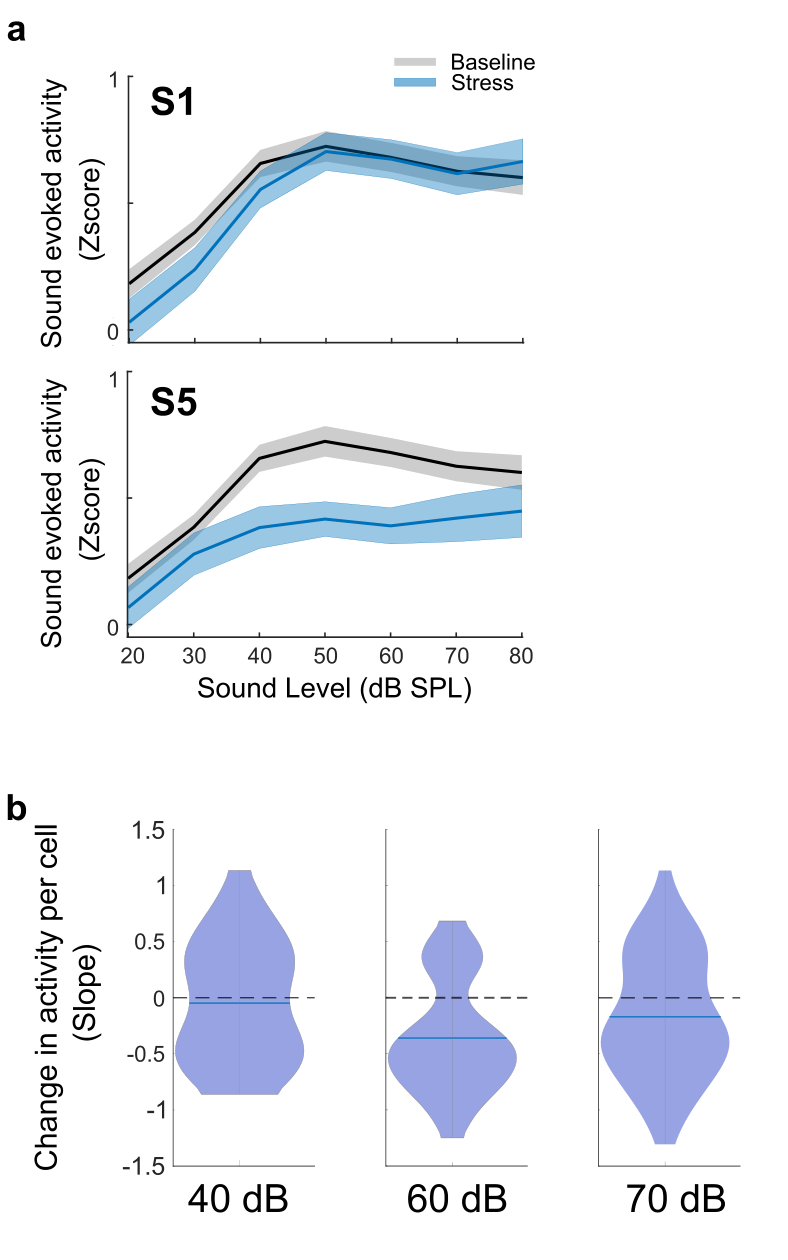

Supplement: S4 Fig — (a) Change of tone-evoked activity over time. Comparison of tone-evoked activity after a day and 5 days of stress to baseline. On the initial day of stress, there was no significant reduction in activity, but as the stressor became chronic, the reduction increased (mean ± SE, 1-way ANOVA, condition S1 F = 1.9, p = 0.16, S5 F = 27.5, p = 1.7 × 10^−07^, nested ANOVA (mouse nested within session) condition S1 F = 2.8, p = 0.09, S5 F = 32.7, p = 1.3 × 10^−08^). (b) Change in activity across sessions per tracked cell in response to 40, 60, and 70 dB white noise. We found a negative slope in most cells especially for mid-intensities, indicating a decrease in activity as the stress becomes chronic (t test for 40 dB p = 0.36, for 60 dB p = 9.3 × 10^−12^, and for 70 dB p = 0.00750). Source data for this figure can be found at: https://www.ebi.ac.uk/biostudies/studies/S-BSST1689754. (TIFF) [file pbio.3003012.s004.tiff]

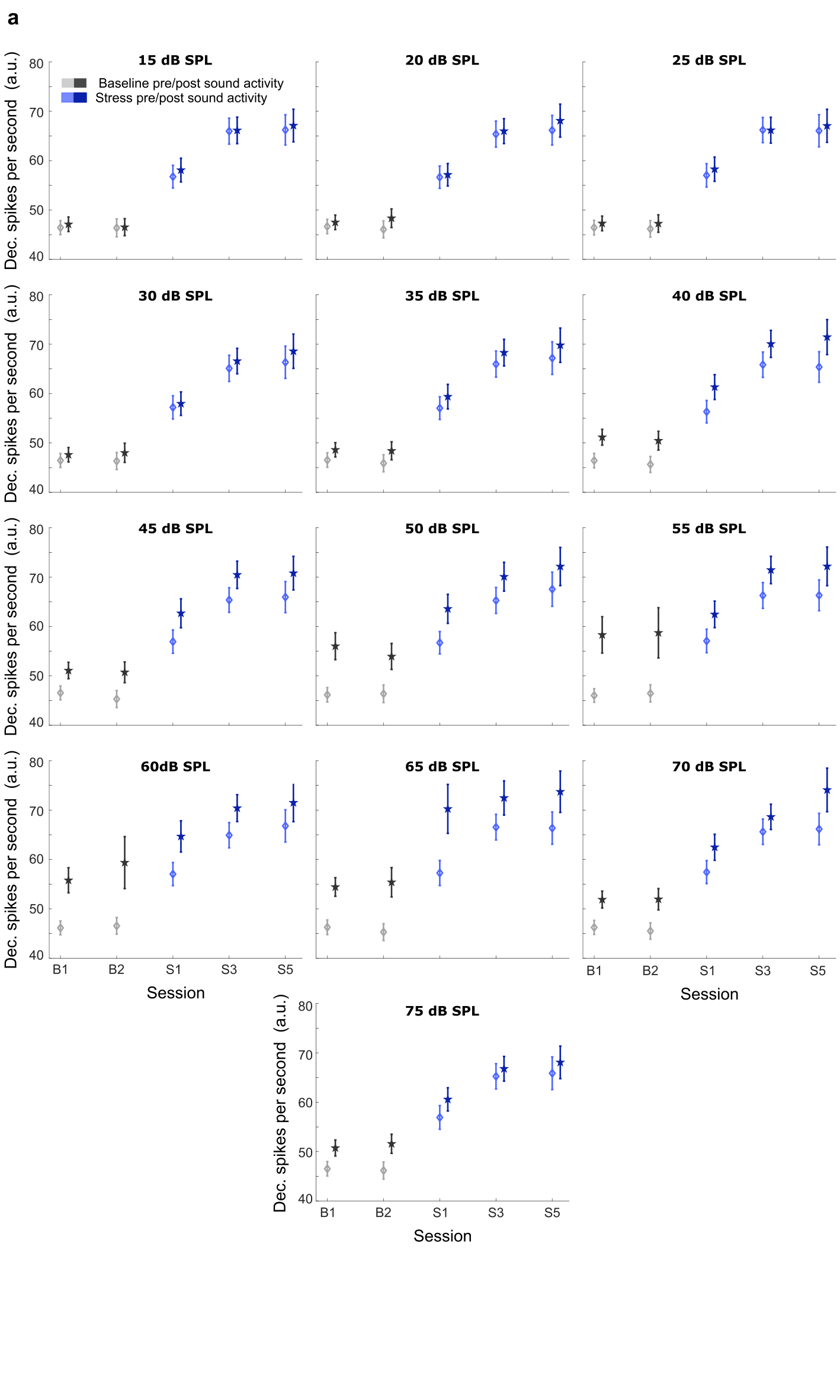

Supplement: S5 Fig — Deconvolved spikes in the pre- and post-sound periods (soft and dark colors accordingly) for PPy cells at different sound intensities in the baseline (gray/black) and stress (light blue/blue) sessions. The pre-sound window was defined as the average activity 300 ms before the sound, and the post-sound period as the average activity 300 ms after the sound’s onset. Values indicate mean ± SE. Source data for this figure can be found at: https://www.ebi.ac.uk/biostudies/studies/S-BSST1689754. (TIFF) [file pbio.3003012.s005.tiff]

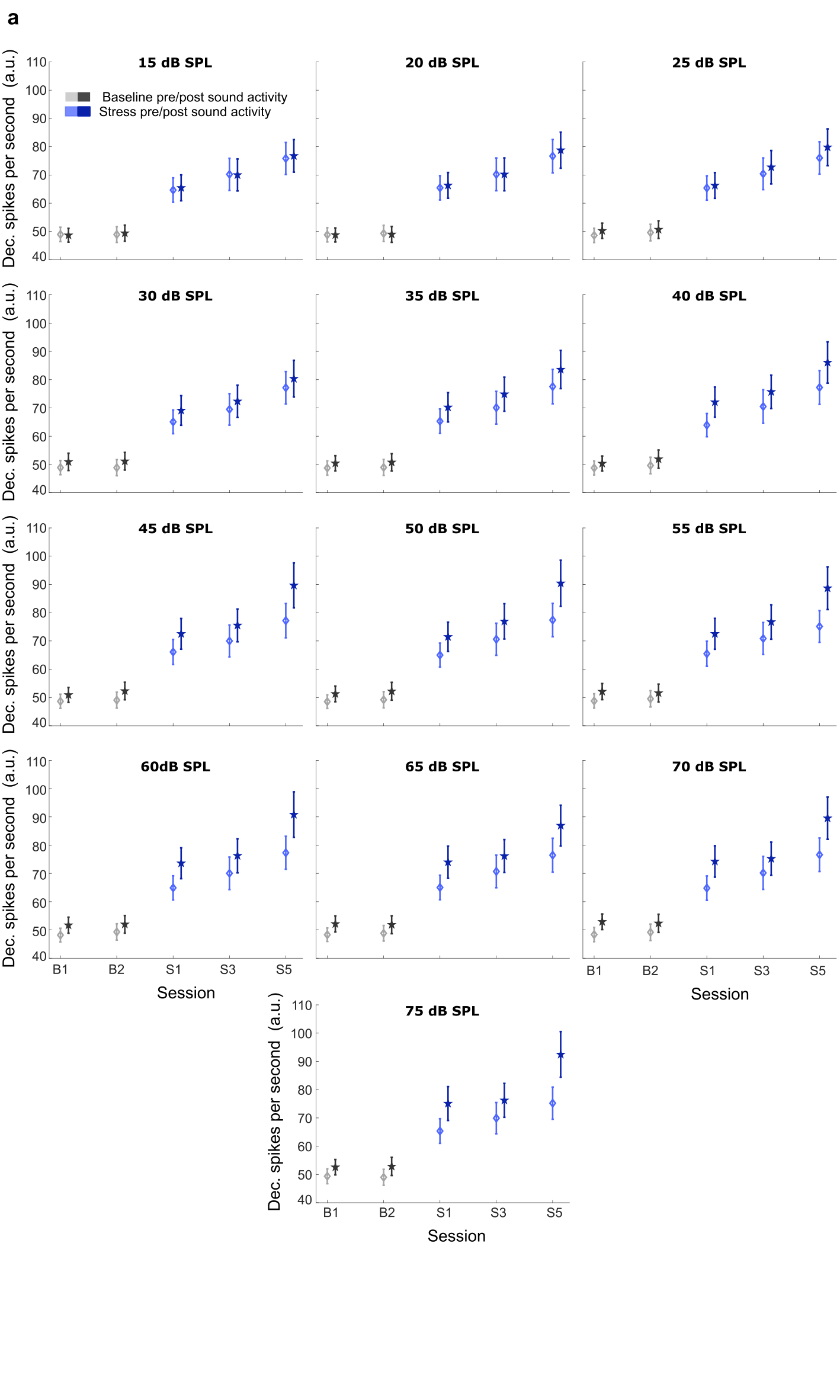

Supplement: S6 Fig — Same as S4 Fig but for SST cells. Deconvolved spikes in the pre- and post-sound periods (soft and dark colors accordingly) for SST cells at different sound intensities in the baseline (gray/black) and stress (light blue/blue) sessions. The pre-sound window was defined as the average activity 300 ms before the sound, and the post-sound period as the average activity 300 ms after the sound’s onset. Values indicate mean ± SE. Source data for this figure can be found at: https://www.ebi.ac.uk/biostudies/studies/S-BSST1689754. (TIFF) [file pbio.3003012.s006.tiff]

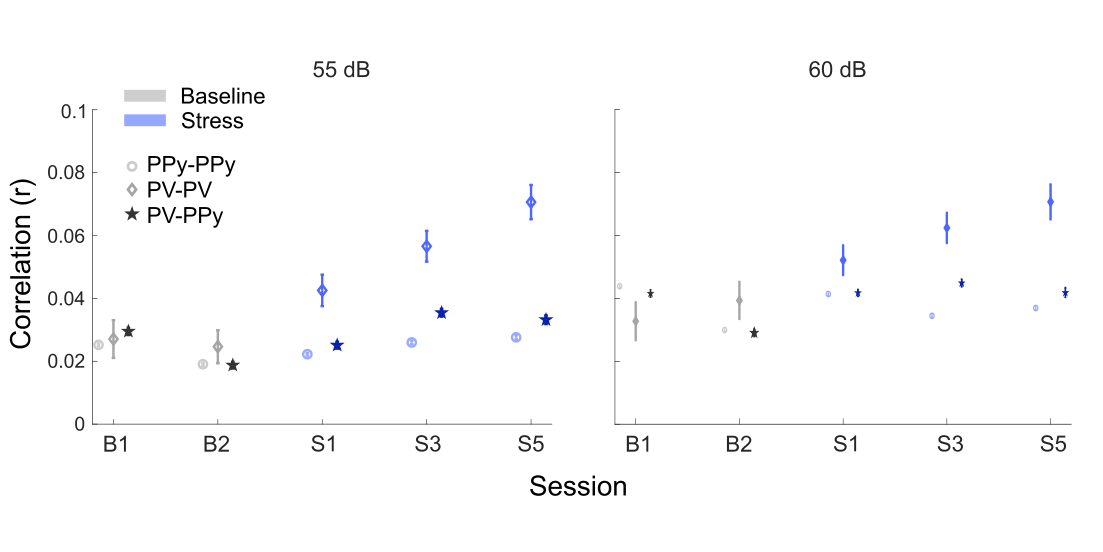

Supplement: S7 Fig — (a) Noise correlations between the normalized activity of pairs of PPy cells, PV cells, and a combination of both in the 500 ms surrounding the noise stimulus presentation at 55 and 60 dB SPL across the different sessions. We found an increase in noise correlations for all types of pairs (2-way ANOVA cell type, session F = 9.5 and 12.2, p = 2.4 × 10^−13^ and 1.3 × 10^−17^, 55 and 60 dB, accordingly). The increase in noise correlations was particularly striking for PV-PV pairs; it was minor the first day of stress but increased as the stressor became chronic (post hoc B1 vs. S3 p = 0.004, B1 vs. S5 p = 2.2 × 10^−07^, B2 vs. S3 p = 3.3 × 10^−04^, B2 vs. S5 p = 3.3 × 10^−09^, all Bonferroni corrected). Source data for this figure can be found at: https://www.ebi.ac.uk/biostudies/studies/S-BSST168975. (TIFF) [file pbio.3003012.s007.tiff]
